# A potent inhibitor of PAI-1, MDI-2517, mitigates disease severity in a preclinical systemic sclerosis model

**DOI:** 10.1172/jci.insight.195005

**Published:** 2026-02-17

**Authors:** Enming J. Su, Pei-Suen Tsou, Mark Warnock, Natalya Subbotina, Kris Mann, Sirapa Vichaikul, Alyssa Rosek, Lisa Leung, Xianying Xing, Enze Xing, Olesya Plazyo, Rachael Bogle, Lam C. Tsoi, Cory D. Emal, Dinesh Khanna, John Varga, Thomas H. Sisson, Johann E. Gudjonsson, Daniel A. Lawrence

**Affiliations:** 1 Division of Cardiovascular Medicine, Department of Internal Medicine;; 2 Scleroderma Program, Division of Rheumatology, Department of Internal Medicine;; 3 Division of Pulmonary and Critical Care Medicine, Department of Internal Medicine;; 4 Department of Molecular Integrative Physiology; and; 5 Department of Dermatology; University of Michigan Medical School, Ann Arbor, Michigan, USA.; 6 Department of Chemistry, Eastern Michigan University, Ypsilanti, Michigan, USA.

**Keywords:** Autoimmunity, Pulmonology, Fibrosis, Rheumatology

## Abstract

Systemic sclerosis (SSc) is a complex and heterogeneous condition characterized by progressive fibrosis in multiple organs. Recent studies implicate plasminogen activator inhibitor-1 (PAI-1) in the pathogenesis of SSc, and PAI-1 is considered as a potential target for therapy. Here, using single-cell and spatial RNA-seq analysis of skin biopsies from 18 healthy individuals and 22 SSc patients, we found elevated PAI-1 colocalizing to myofibroblasts with enriched extracellular matrix–associated biological processes. Treatment of SSc dermal fibroblasts with the small-molecule PAI-1 inhibitor MDI-2517 reduced the expression of the profibrotic markers *COL1A1* and *ACTA2*. To investigate the therapeutic potential of MDI-2517, we evaluated its efficacy in reducing fibrosis in a preclinical model of SSc. Treatment of mice with MDI-2517 significantly reduced both skin and lung fibrosis and was superior to treatment with either pirfenidone or mycophenolate mofetil. Additionally, MDI-2517 attenuated weight loss and significantly reduced the expression of key profibrotic markers. Compared with tiplaxtinin, another PAI-1 inhibitor previously shown to be effective in a model of SSc, MDI-2517 was found to have superior efficacy at a 10-fold lower dose. These findings highlight the role of PAI-1 in the pathogenesis of SSc, and the potential of MDI-2517 for the treatment of SSc.

## Abstract 2

These findings highlight the role of PAI-1 in the pathogenesis of Systemic sclerosis (SSc) , and the potential of MDI-2517 for the treatment of SSc.

## Graphical abstract

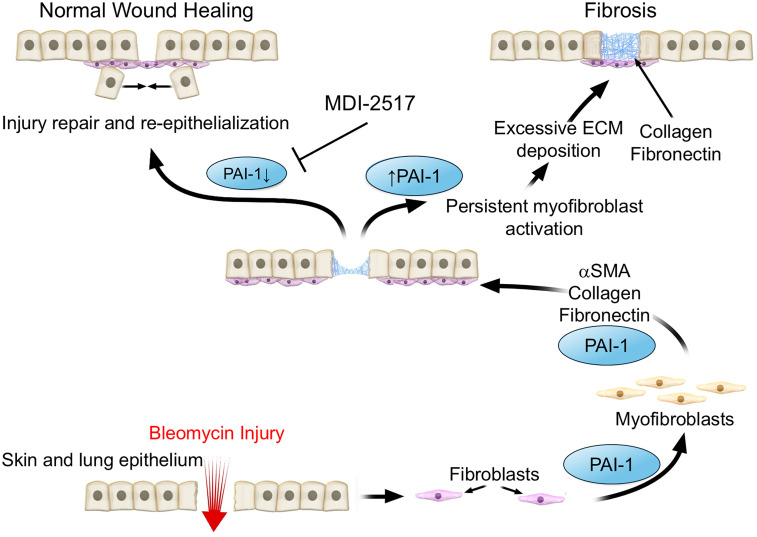

## Introduction

Systemic sclerosis (SSc), also called scleroderma, is a chronic autoimmune disorder characterized by fibrosis (thickening and hardening) of the skin, blood vessels, and internal organs. This condition primarily affects the connective tissue, resulting in widespread inflammation and the overproduction of collagen (1, 2). SSc is associated with a range of symptoms, including skin tightening, joint pain, organ dysfunction, and vascular complications. Clinically progressive interstitial lung disease (ILD) occurs in approximately 40% of SSc cases and is the leading cause of death in SSc (3–7). There is no known cure for SSc, and current treatments aim to manage symptoms, slow disease progression, and improve quality of life (8). Treatment options involve a combination of pharmacological and non-pharmacological interventions. Pharmacotherapies focus on controlling inflammation, alleviating symptoms, and targeting specific manifestations of the disease. Immunosuppressive medications, such as methotrexate, mycophenolate mofetil (MMF), and cyclophosphamide, are used to modulate the immune response and reduce inflammation (9). For the treatment of SSc-ILD, the two approved therapies, nintedanib and tocilizumab, only slow, but do not stop or reverse, the decline in pulmonary function (10).

In recent years, targeted therapies have shown promise in managing SSc. Studies have identified potential molecular targets and pathways that play a crucial role in the pathogenesis of the disease. One such target is plasminogen activator inhibitor-1 (PAI-1), a protein involved in the regulation of fibrinolysis, extracellular matrix (ECM) remodeling, and wound healing (11–15). PAI-1, encoded by the *SERPINE1* gene, is elevated in SSc patients, and is thought to contribute to the excessive accumulation of collagen, leading to tissue fibrosis (16, 17). PAI-1 serves as the physiological inhibitor of tissue-type and urokinase-type plasminogen activator (tPA and uPA). Under physiological conditions, PAI-1 plays an important role in regulating fibrinolysis and wound healing. However, excessive PAI-1 activity has been strongly associated with fibrotic diseases, including pulmonary fibrosis. Animal models of pulmonary fibrosis have indicated that inhibiting PAI-1 could be an effective therapeutic strategy for this debilitating condition (18, 19).

In models of SSc, two preclinical studies have targeted PAI-1 with either the small molecule tiplaxtinin (20) or a monoclonal antibody against PAI-1 (11), and both showed reductions in skin fibrosis. In another study using transgenic mice overexpressing the transcription factor Snail in keratinocytes, the mice developed an SSc-like phenotype that was largely normalized by systemic PAI-1 deficiency (21). Together, these studies suggest that PAI-1 is an important mediator of fibrosis development in SSc. MDI-2517 is a more effective analog of a new class of small-molecule inhibitors targeting PAI-1 (22), with a unique mechanism of action, and is currently in phase I clinical testing (NCT06453824). MDI-2517 offers the potential of an antifibrotic treatment by moderating the activity of PAI-1, which contributes to the pathogenesis of SSc. In the studies presented here, we sought to evaluate PAI-1 expression in patients with SSc and to assess MDI-2517 in a model of SSc for efficacy to reduce fibrogenic progression, collagen accumulation, and overall disease severity in both skin and lung. Our results provide important insight into the therapeutic potential of MDI-2517 for the treatment of SSc with ILD.

## Results

### Expression of PAI-1 in human SSc skin samples.

**Figure 1:**
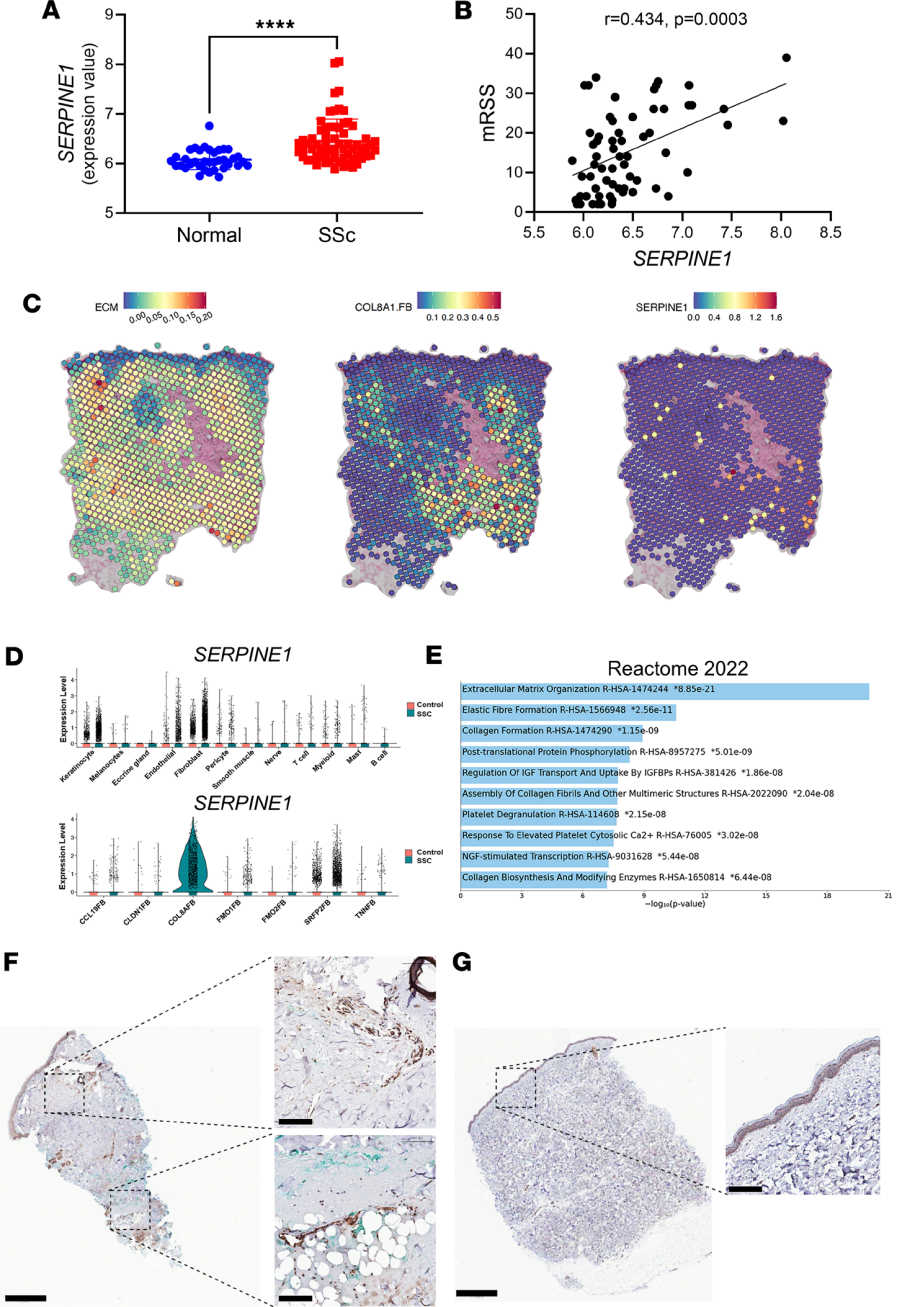
Expression of PAI-1 in SSc skin. (**A**) Comparison of *SERPINE1* expression in SSc and healthy control skin biopsy samples from the GEO dataset (GSE58095). (**B**) Correlation analysis of modified Rodnan skin scores (mRSS) versus *SERPINE1* expression from the data in **A**. (**C**) Enriched signature for expression of extracellular matrix (ECM) genes along with expression of the myofibroblast marker *COL8A1*, and *SERPINE1* (PAI-1) expression in SSc skin on the 10x Visium spatial platform (data representative of *n* = 4). (**D**) Violin plots of *SERPINE1* expression in controls versus SSc skin across single-cell RNA-seq data from different cellular populations (top) and fibroblast subsets (bottom) (*n* = 18 healthy controls, *n* = 22 SSc patients). (**E**) Enriched biological processes in *SERPINE1^+^* versus *SERPINE1^–^* *COL8A1* myofibroblasts. (**F**) IHC of PAI-1 in SSc skin biopsy (data representative of *n* = 6). (**G**) IHC of PAI-1 in skin biopsy from a healthy volunteer. Scale bars: 500 μm on overall biopsy, 50 μm on the insets. ****P < 0.0001 by 2-tailed Mann-Whitney test in **A**. *P* = 0.0003 by Spearman’s *r* correlation test in **B**.

Comparison of *SERPINE1* expression in skin biopsies from 36 normal individuals versus 66 SSc patients from the Gene Expression Omnibus (GEO) dataset (GSE58095) demonstrated a significant increase in *SERPINE1* expression in the skin of patients that was strongly correlated with their modified Rodnan skin score (Figure 1, A and B). We next used single-cell RNA-seq analyses of skin biopsies from 18 healthy controls and 22 SSc patients along with spatial sequencing of lesional SSc skin from 4 subjects that we previously reported (23) to assess *SERPINE1* expression. We observed a marked overlap in the expression of *SERPINE1* and genes involved in ECM remodeling and myofibroblast phenotype (as defined by *COL8A1* expression) (Figure 1C and Supplemental Figure 1; supplemental material available online with this article; https://doi.org/10.1172/jci.insight.195005DS1). This finding was verified by identification of increased *SERPINE1* expression in cell types obtained from lesional SSc versus healthy control skin. Specifically, we found upregulated expression of *SERPINE1* in SSc fibroblasts and, to a lesser extent, in SSc endothelial cells and keratinocytes relative to their control counterparts (Figure 1D, top). When assessed across fibroblast subsets, the most prominent expression of *SERPINE1* was observed in *COL8A1* myofibroblasts (Figure 1D, bottom). We further compared enriched biological processes (Reactome) in *SERPINE1^+^* versus *SERPINE1^–^* myofibroblasts and identified an enrichment of biological processes related to ECM, including ECM organization, collagen formation, and assembly of collagen fibrils (Figure 1E) (see Supplemental Table 1 for the gene components of our ECM module score). The presence of PAI-1 protein in SSc skin was validated by immunohistochemistry, whereas little PAI-1 staining is observed in skin from a healthy control (Figure 1, F and G).

### Downregulation of profibrotic biomarkers by PAI-1 inhibition in isolated SSc skin fibroblasts.

**Figure 2:**
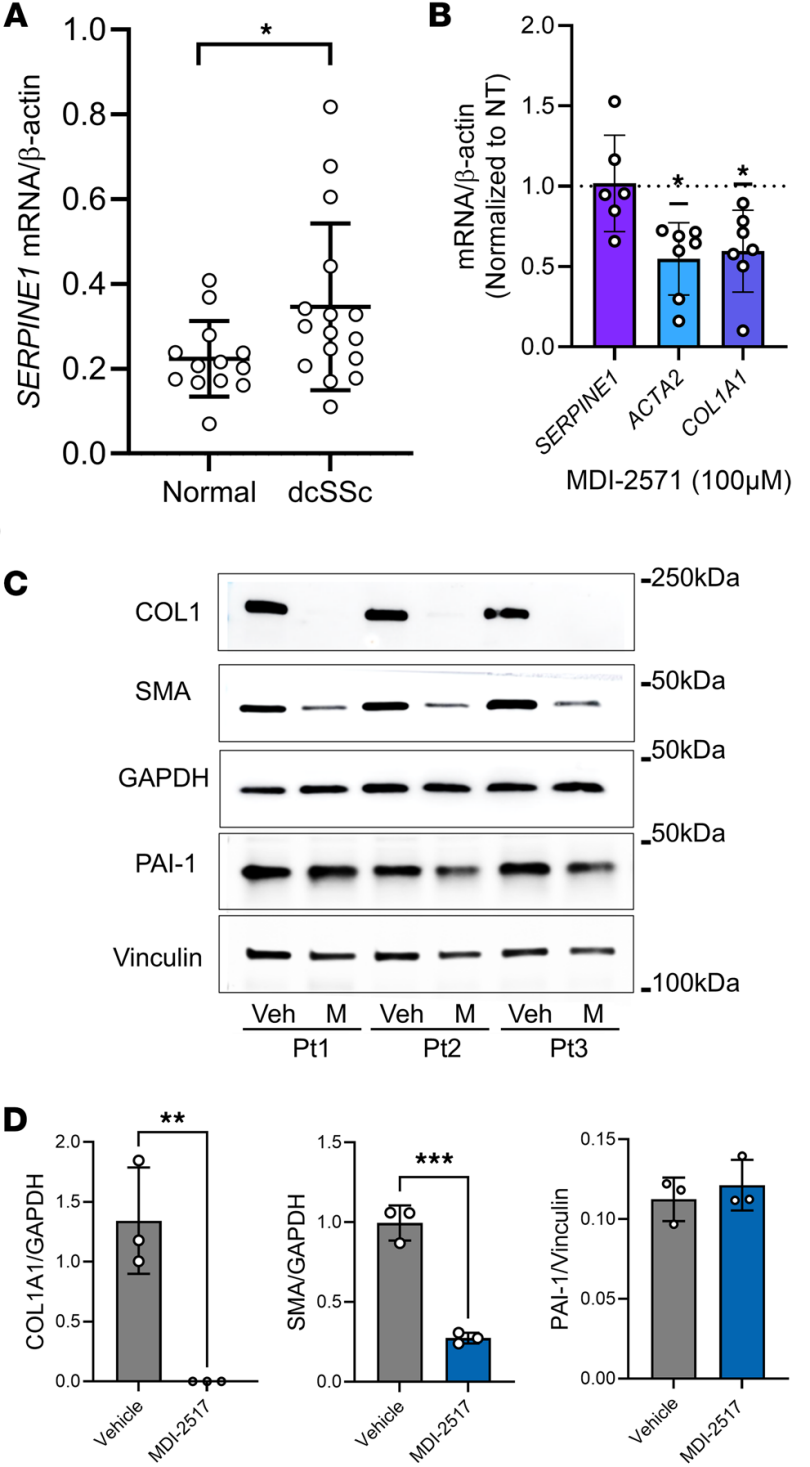
Downregulation of profibrotic markers in human SSc fibroblasts by MDI-2517. Dermal fibroblasts were isolated from punch biopsies from the distal forearm of healthy volunteers and diffuse cutaneous SSc (dcSSc) patients. (**A**) qPCR of PAI-1 (*SERPINE1*) mRNA expression in fibroblasts from normal skin and from SSc patients. (**B**) Downregulation of mRNA expression of the profibrotic markers *ACAT2* and *COL1A1* with MDI-2517 treatment. (**C**) Western blot analysis shows reduction in COL1, αSMA, and PAI-1 protein in SSc fibroblasts with and without MDI-2517 treatment. (**D**) Quantification of the Western blots in **C**; αSMA and COL1 are shown as the ratio to GAPDH, and PAI-1 as the ratio to vinculin. Data are shown as mean ± SD; *n* is indicated in each figure by the individual data points (3 to 17); *P < 0.05, **P < 0.01, ***P < 0.001 by 2-tailed Mann-Whitney test in **A**, 1-sample Wilcoxon’s test in **B**, and 2-tailed *t* test in **D**.

Consistent with the single-cell sequencing data above, we found that in dermal fibroblasts collected from diffuse cutaneous SSc patients, there was a significant increase in PAI-1 expression compared with expression in dermal fibroblasts from healthy volunteers (Figure 2A). To examine the potential antifibrotic action of PAI-1 inhibition in SSc dermal fibroblasts, we measured gene expression of the fibrotic biomarkers α-smooth muscle actin (αSMA; gene *ACTA2*) and collagen 1A1 (gene *COL1A1*) with and without MDI-2517 treatment. We also examined the effect of MDI-2517 on the expression of *SERPINE1*. These data demonstrated that MDI-2517 treatment induced an approximately 50% reduction in both *ACTA2* and *COL1A1* expression in comparison with vehicle-treated cells but did not inhibit *SERPINE1* expression (Figure 2B and Supplemental Figure 2). The lack of a direct inhibition of *SERPINE1* expression is consistent with the known mechanism of action of this class of PAI-1 inhibitors that act directly on PAI-1 protein to inhibit its function but have not been shown to affect its expression (22). The reduction in the fibrotic response without reducing PAI-1 expression was further confirmed by immunoblotting for the protein products αSMA, COL1, and PAI-1 in SSc dermal fibroblasts treated with or without MDI-2517 (Figure 2, C and D).

### Oral administration of MDI-2517 reduces PAI-1 in skin, and systemic PAI-1 activity.

**Figure 3:**
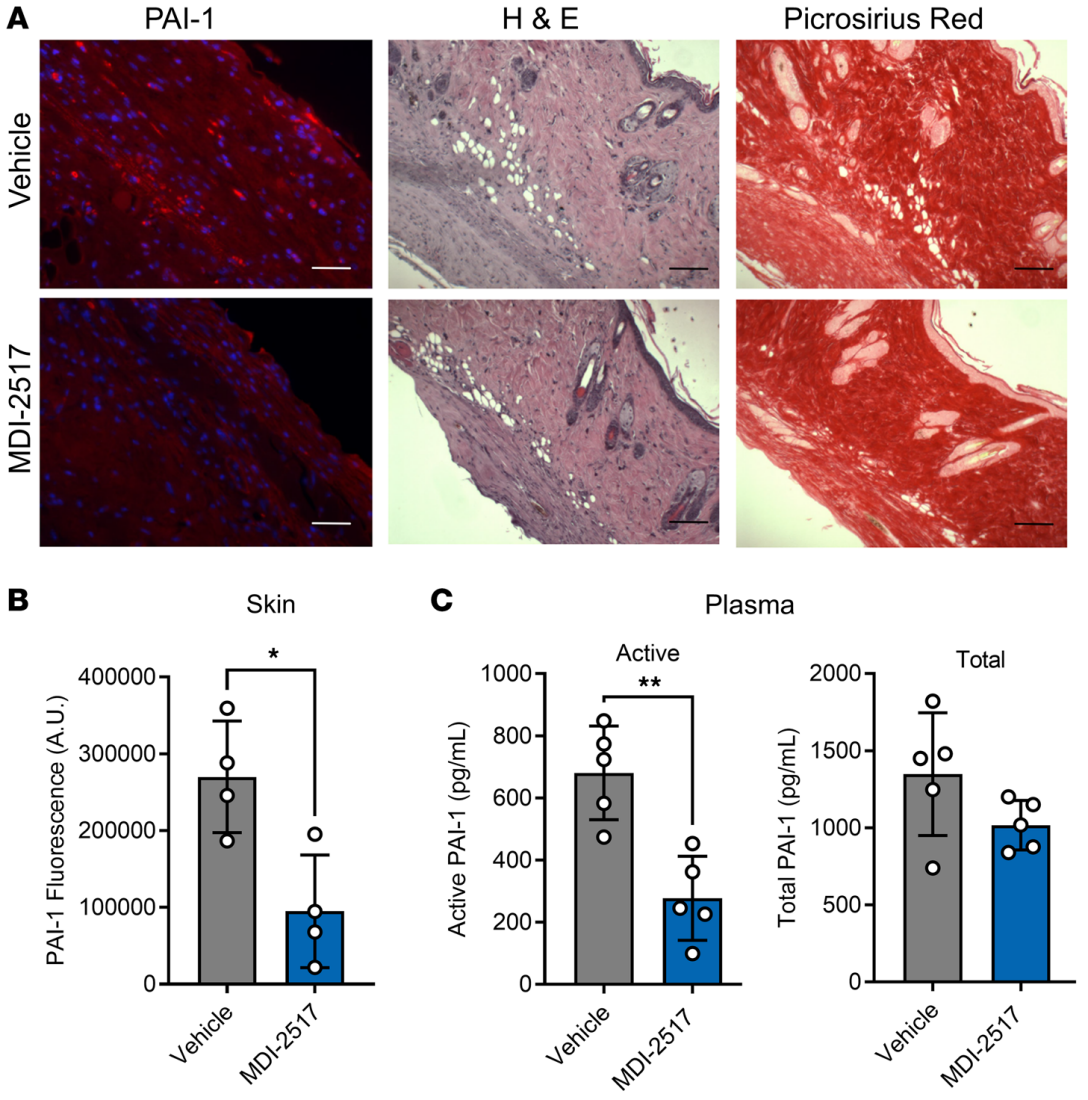
Target engagement of MDI-2517. Twelve-week-old male C57BL/6J mice were subcutaneously implanted with osmotic pumps that delivered 100 U/kg (total) of bleomycin over 7 days. The pumps were then removed, and at that time the mice were placed on chow containing 0 mg/kg (Vehicle) or MDI-2517 at 500 mg/kg chow. On day 28, skin tissue and plasma and were collected. PAI-1 antigen in skin was quantified by immunofluorescence microscopy; images were acquired using the same settings and taken in comparable regions for each skin sample. For quantification, 5 areas of interest per skin sample were analyzed (*n* = 4 mice per treatment group), and the area of antibody immunoreactivity above a set threshold was measured and averaged for each animal using ImageJ. (**A**) Immunofluorescent staining of PAI-1 (red) and DAPI nuclear stain (blue), hematoxylin and eosin (H&E), and Picrosirius red staining. (**B**) Quantification of the relative PAI-1 antigen in skin of the untreated and MDI-2517–treated mice. (**C**) Active and total PAI-1 in plasma. Scale bars: 100 μm. Data are shown as mean ± SD; *n* is indicated in each figure by the individual data points (4 to 5); *P < 0.05 in **B**, **P < 0.01 in **C**, by 2-tailed *t* test.

Subcutaneous bleomycin administration to induce fibrosis is commonly used as a model of SSc in preclinical studies to evaluate the efficacy of antifibrotic treatments (24). For our studies, bleomycin (100 U/kg) was administered subcutaneously via an osmotic pump over a period of 7 days to induce systemic fibrosis. After 7 days, the pumps were removed, and animals were then placed on control chow or chow containing MDI-2517 (500 mg of MDI-2517 per kg of chow) for an additional 3 weeks. This formulation was based on a preliminary dose finding study indicating that 500 mg of MDI-2517 per kg of chow was a maximally effective dose at reducing skin thickening (Supplemental Figure 3). Based on the average consumption of 10–15 g of chow per day per 100 g of body weight for mice (25), this drug concentration is expected to result in an approximate daily dose of MDI-2517 of 60 mg/kg. After this treatment we examined target engagement by MDI-2517 in both skin and plasma. PAI-1 protein in skin and systemic PAI-1 antigen and activity in plasma were quantified on day 28. PAI-1 protein levels in skin were determined by immunofluorescent staining (Figure 3, A and B), and PAI-1 antigen and activity in plasma were analyzed by a multiplex Luminex assay as previously described (26) (Figure 3C). Similarly to human skin from a healthy control (Figure 1G), naive mouse skin displayed little PAI-1 immunofluorescence (Supplemental Figure 4). In contrast, there was notable PAI-1 protein in mouse skin following bleomycin exposure that was significantly reduced by approximately 65% in MDI-2517–treated mice in comparison with vehicle treatment (Figure 3, A and B). Likewise, the activity of PAI-1 in plasma showed a similar 56% reduction in MDI-2517–treated animals, while total PAI-1 antigen showed a non-significant reduction (Figure 3C).

### MDI-2517 treatment is more efficacious than pirfenidone or mycophenolate mofetil at reducing bleomycin-induced skin fibrosis in the bleomycin model of SSc.

**Figure 4:**
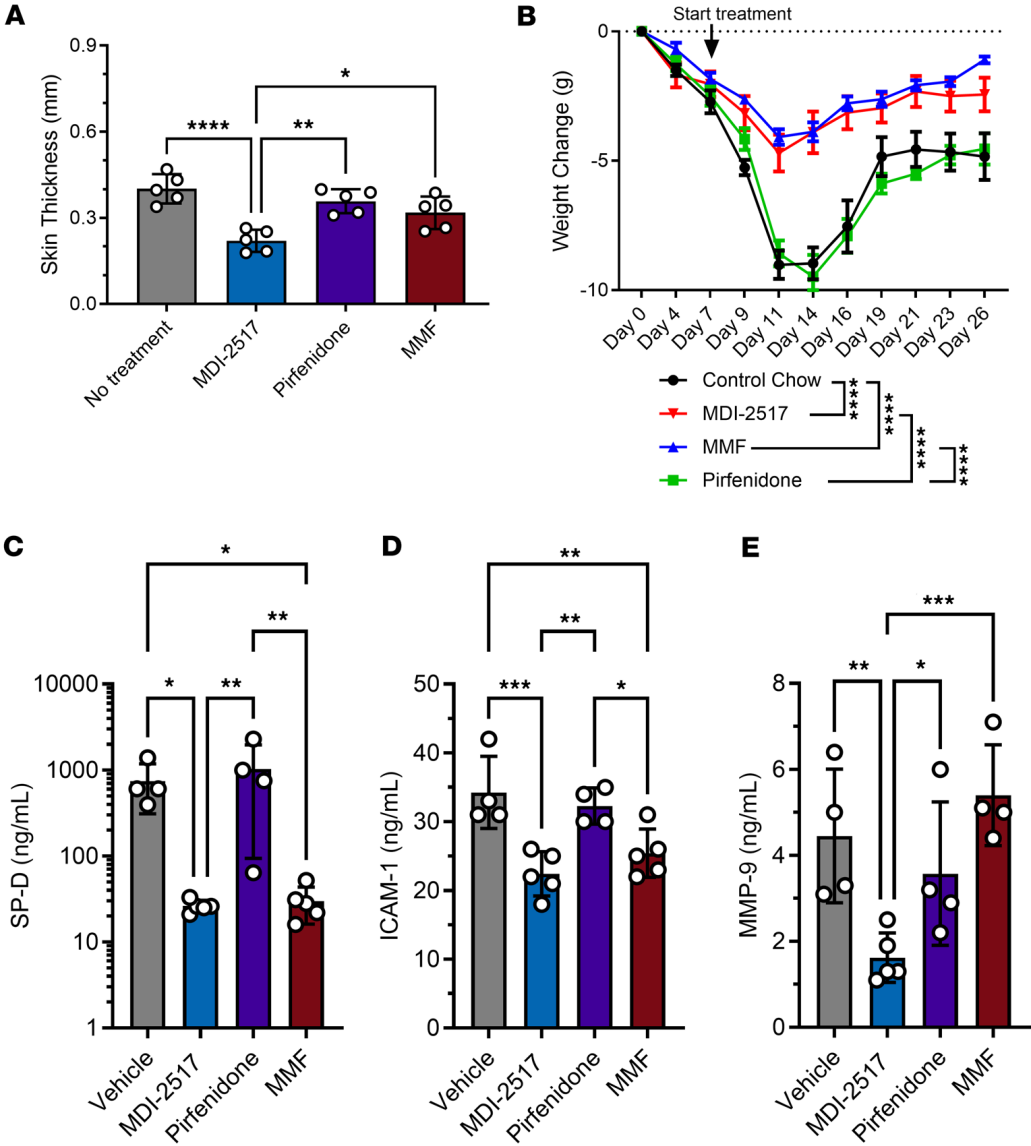
Comparator study of MDI-2517, pirfenidone, and mycophenolate mofetil. Twelve-week-old male C57BL/6J mice were subcutaneously implanted with osmotic pumps that delivered 100 U/kg (total) of bleomycin over 7 days. The pumps were then removed, and at that time the mice were placed on treatment chows (drug concentration in chow: vehicle, 0 mg/kg; MDI-2517 at 500 mg/kg; pirfenidone at 1,000 mg/kg; mycophenolate mofetil [MMF] at 1,000 mg/kg). (**A**) On day 28, skin thickness was determined at multiple locations by skin pinch with calipers; then mice were sacrificed and plasma and skin tissues collected. (**B**) Body weight changes monitored approximately every 3 days. (**C**–**E**) Biomarkers measured by antibody-based Luminex multiplex assay. Data are shown as mean ± SD; *n* is indicated in each figure by the individual data points (4 to 5); *P < 0.05, **P < 0.01, ***P < 0.001, ****P < 0.0001 by 1-way ANOVA in **A** and **C**–**E** and by a repeated-measures mixed-effects model in **B**.

To compare the therapeutic efficacy of MDI-2517 to that of clinically administered antifibrotic and immunosuppressive drugs, including the current first-line standard of care mycophenolate mofetil (MMF) (27), systemic fibrosis was induced with bleomycin as above, and the mice were placed on chow containing either MDI-2517 (500 mg/kg of chow), MMF (1,000 mg/kg of chow), or pirfenidone (1,000 mg/kg of chow). Pirfenidone is approved for the treatment of idiopathic pulmonary fibrosis and has been investigated in clinical trials for SSc (28). Compared with either MMF or pirfenidone, MDI-2517 showed superior efficacy in reducing skin thickness with over 50% reduction in comparison with vehicle control. In contrast, MMF treatment reduced skin thickness by approximately 15% (*P* = 0.057), while pirfenidone had no effect (Figure 4A). Further, both MDI-2517– and MMF-treated animals showed attenuated weight loss compared with vehicle or pirfenidone treatment (Figure 4B). Known biomarkers of SSc were also examined, and these data demonstrated that MDI-2517 treatment reduced key biomarkers associated with disease progress in SSc patients (Figure 4, C–E). The biomarkers examined were serum surfactant protein-D (SP-D), a marker associated with the decline of forced vital capacity; intercellular adhesion molecule 1 (ICAM-1), a marker known for regulating leukocyte recruitment from circulation to sites of inflammation; and MMP-9, a marker of fibrogenic remodeling during the progression of skin sclerosis in SSc. MDI-2517 showed superior efficacy in reducing all 3 biomarkers (SP-D, ICAM-1, and MMP-9) compared with pirfenidone and was superior to MMF in reducing MMP-9 (Figure 4, C–E).

### Comparator study of MDI-2517 and MDI-2517 plus MMF combination in the bleomycin model of SSc.

**Figure 5:**
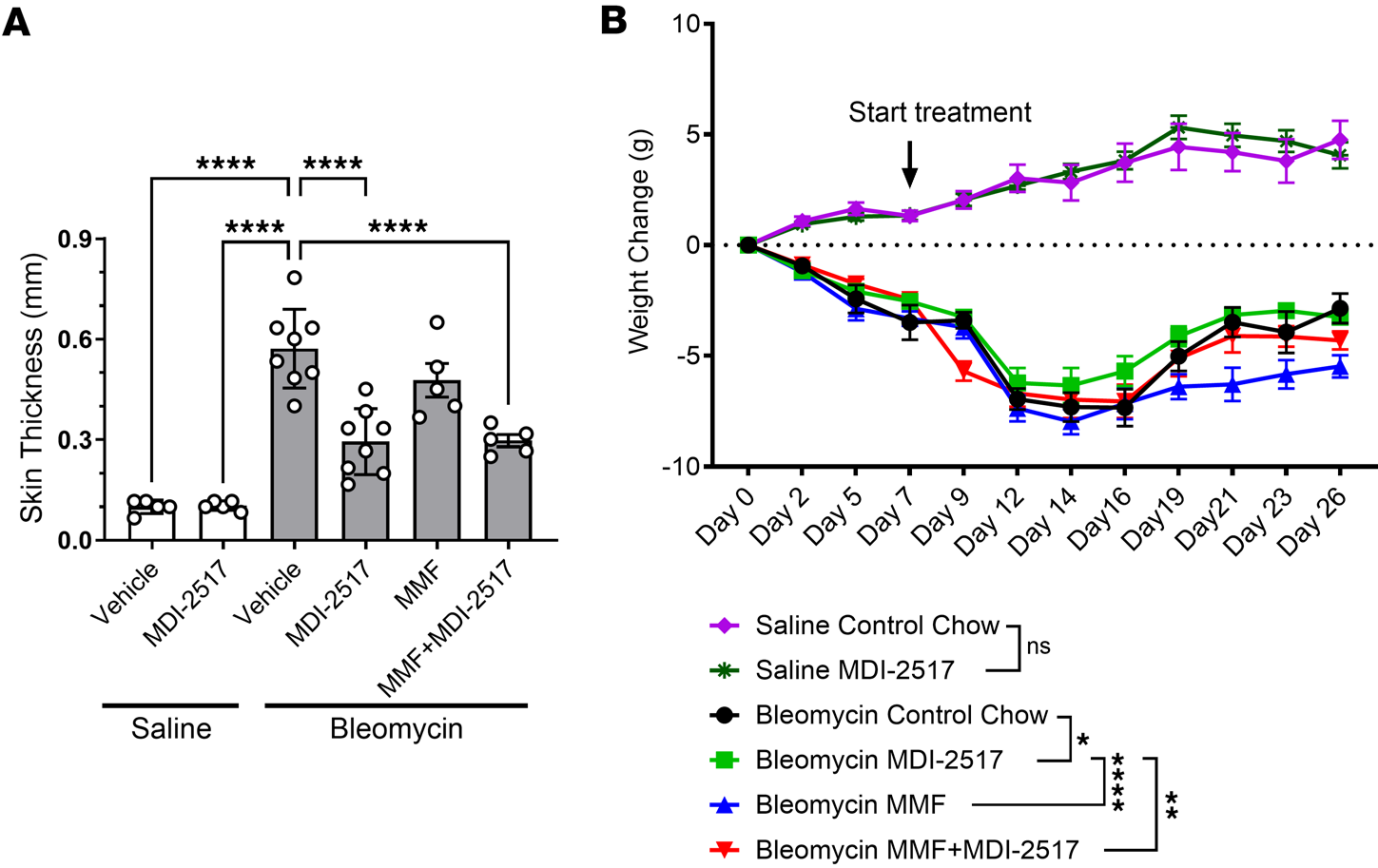
Comparator study of MDI-2517, MMF, and MMF plus MDI-2517. Twelve-week-old male C57BL/6J mice were subcutaneously implanted with osmotic pumps that delivered 100 U/kg (total) of bleomycin over 7 days. The pumps were then removed, and at that time the mice were placed on treatment chows (drug concentration in chow: vehicle, 0 mg/kg; MDI-2517 at 500 mg/kg; MMF at 1,000 mg/kg; or combined MDI-2517 at 500 mg/kg and MMF at 1,000 mg/kg). (**A**) On day 28, skin thickness was determined at multiple locations by skin pinch with calipers; then mice were sacrificed and tissues collected. (**B**) Body weight changes monitored approximately every 3 days. Data are shown as mean ± SD; *n* is indicated in each figure by the individual data points (5 to 8); *P < 0.05, **P < 0.01, ****P < 0.0001 by 1-way ANOVA in **A** and by a repeated-measures mixed-effects model in **B**.

Since MMF is the standard of care for SSc patients, we examined whether MDI-2517 would have benefit in combination with MMF in attenuating bleomycin-induced skin fibrosis. As a control, pumps filled with saline were also implanted in 2 separate cohorts; one cohort was treated with control chow and another with chow containing MDI-2517 (500 mg/kg of chow). Compared with MMF alone or the control group, treatment with MDI-2517 or a combination of MDI-2517 plus MMF showed superior efficacy in reducing skin thickness with approximately 50% reduction compared with control chow, and similar to the results above, MMF alone resulted in a non-significant 16% reduction in skin thickness (Figure 5A). Additionally, MDI-2517–treated animals showed better attenuation in weight loss compared with vehicle- and MMF-treated animals (Figure 5B). MDI-2517 treatment in the absence of the bleomycin insult did not affect dermal thickness or weight (Figure 5, A and B).

**Figure 6:**
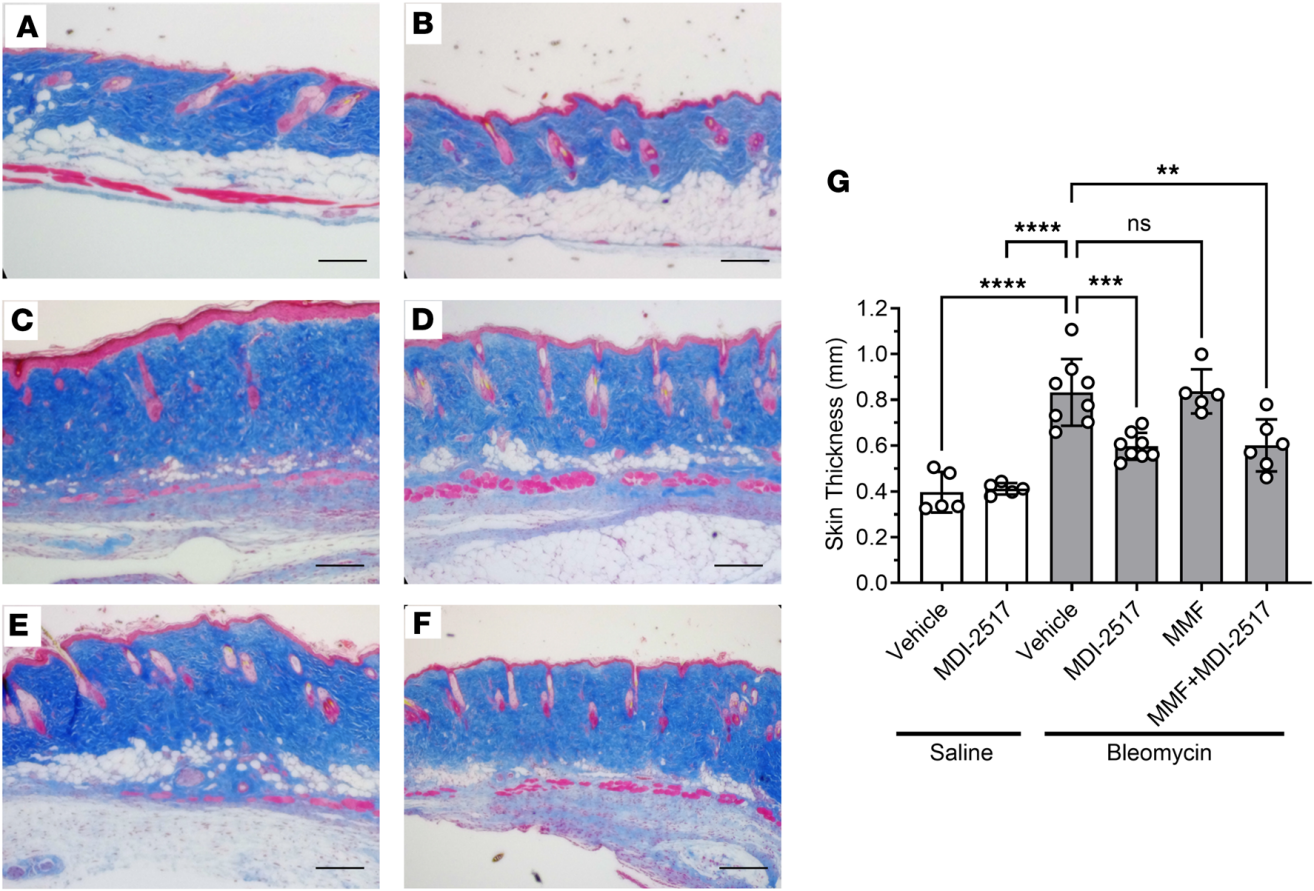
Skin trichrome staining from the comparator study of MDI-2517, MMF, and combined MMF plus MDI-2517. Twelve-week-old male C57BL/6J mice were subcutaneously implanted with osmotic pumps that delivered 100 U/kg (total) of bleomycin or saline over 7 days. The pumps were then removed, and at that time the mice were placed on treatment chows (drug concentration in chow: vehicle, 0 mg/kg; MDI-2517 at 500 mg/kg; MMF at 1,000 mg/kg; or combined MDI-2517 at 500 mg/kg and MMF at 1,000 mg/kg). On day 28, skin tissues were prepared for histological analysis by Masson’s trichrome stain. (**A**) Saline pump control chow. (**B**) Saline pump MDI-2517 chow. (**C**) Bleomycin control chow (no treatment). (**D**) Bleomycin MDI-2517 chow. (**E**) Bleomycin MMF chow. (**F**) Bleomycin combined MMF and MDI-2517 chow. (**G**) Quantification of skin thickness from Masson’s trichrome–stained slides. Scale bars: 100 μm. Data are shown as mean ± SD; *n* is indicated in each figure by the individual data points (5 to 8); **P < 0.01, ***P < 0.001, ****P < 0.0001 by 1-way ANOVA.

To confirm skin thickness results measured by the pinch method, skin samples were processed and stained with Masson’s trichrome (Figure 6). Figure 6, A and B, shows the dermal layer of animals that received saline pumps and were treated with control chow or chow containing MDI-2517, respectively. Compared with these control conditions, animals receiving bleomycin pumps and treated with control chow had a much thicker dermal layer (Figure 6C), whereas MDI-2517–treated skin samples showed substantially less collagen deposition and a significantly thinner dermal layer (Figure 6, D and G). Interestingly, the adipose layer also appeared to be better preserved in MDI-2517– and MMF-treated samples, while this layer had almost disappeared in the vehicle controls (compare Figure 6, C and D). MMF-treated animals did not show any improvements in dermal thickness but did show apparent adipose layer preservation (Figure 6E). Finally, animals treated with the combination of MDI-2517 and MMF showed better attenuation of dermal thickening than control- or MMF-only-treated animals (Figure 6, F and G).

### Reduced lung fibrosis by MDI-2517 in the bleomycin model of SSc.

**Figure 7:**
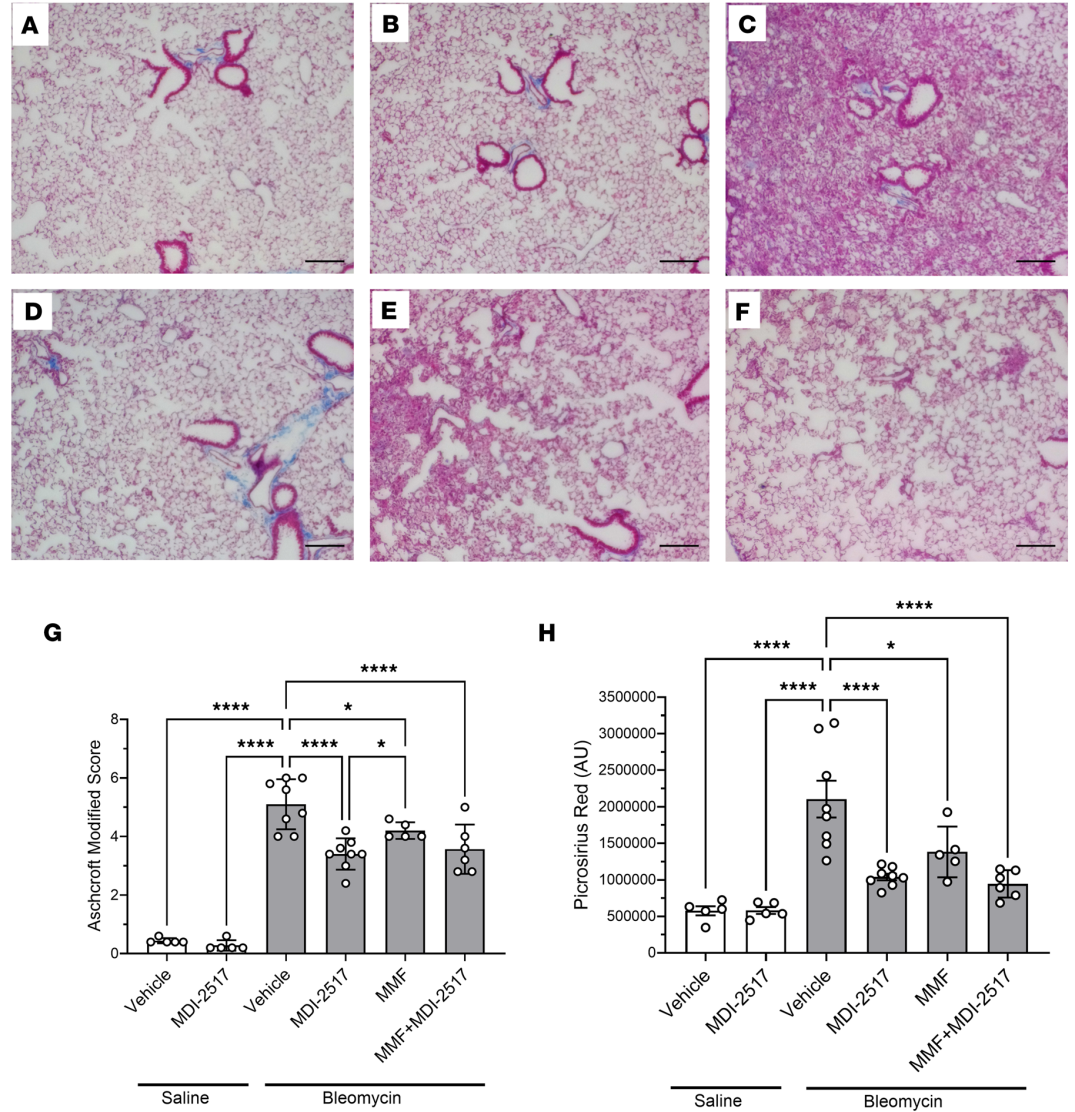
Lung trichrome staining from the comparator study of MDI-2517, MMF, and combined MMF plus MDI-2517. Twelve-week-old male C57BL/6J mice were implanted with osmotic pumps that delivered 100 U/kg (total) of bleomycin or saline over 7 days. The pumps were then removed, and at that time the mice were placed on treatment chows (drug concentration in chow: vehicle, 0 mg/kg; MDI-2517 at 500 mg/kg; MMF at 1,000 mg/kg; or combined MDI-2517 at 500 mg/kg and MMF at 1,000 mg/kg). On day 28, lung tissues were prepared for histological analysis by Masson’s trichrome stain and Picrosirius red stain. (**A**) Saline pump control chow. (**B**) Saline pump MDI-2517 chow. (**C**) Bleomycin control chow (no treatment). (**D**) Bleomycin MDI-2517 chow. (**E**) Bleomycin MMF chow. (**F**) Bleomycin combined MMF and MDI-2517 chow. (**G**) Modified Ashcroft score of lung tissue from Masson’s trichrome–stained slides. (**H**) Picrosirius red quantification from stained slides. Scale bars: 35 μm. Data are shown as mean ± SD; *n* is indicated in each figure by the individual data points (5 to 8); *P < 0.05, ****P < 0.0001 by 1-way ANOVA in **G** and **H**.

Systemic bleomycin treatment causes not only skin fibrosis but also the subsequent development of lung fibrosis. Therefore, lung histology was examined for fibrosis, and Figure 7, A–F, shows closeup images of the fibrotic areas. Similar to the distribution of disease observed in patients with usual interstitial pneumonia, systemic bleomycin exposure induced a pattern of subpleural fibrosis, and this is shown in the low-power images in Supplemental Figure 5. Figure 7, A and B, shows lung sections from control mice that received saline pumps and were treated with control chow or chow containing MDI-2517, respectively. Figure 7, C–F, shows representative histopathological sections from mice receiving a bleomycin pump. In contrast to lungs from bleomycin-injured mice treated with control chow that showed substantial fibrotic changes (Figure 7C), lungs from bleomycin-treated mice that received MDI-2517 showed only mild to moderate fibrotic changes (Figure 7D). Blinded assessment using the modified Ashcroft scoring system for lung fibrosis (Figure 7G) showed that both MDI-2517 and MMF afforded protection. However, MDI-2517–treated mice showed significantly lower Ashcroft scores than mice treated with MMF alone (Figure 7G). Combination treatment of MDI-2517 and MMF resulted in antifibrotic effects comparable to those of MDI-2517 treatment alone. Fibrosis was also analyzed in tissue sections stained with Picrosirius red to visualize collagen (Supplemental Figure 6). The images were then quantified based on the intensities of Picrosirius red staining (Figure 7H), which showed results similar to those obtained with the modified Ashcroft scoring system, with MDI-2517, MMF, and the combination treatment having significantly reduced collagen compared with the vehicle control.

### Comparator study of MDI-2517 versus the PAI-1 inhibitor tiplaxtinin in the bleomycin model of SSc.

**Figure 8:**
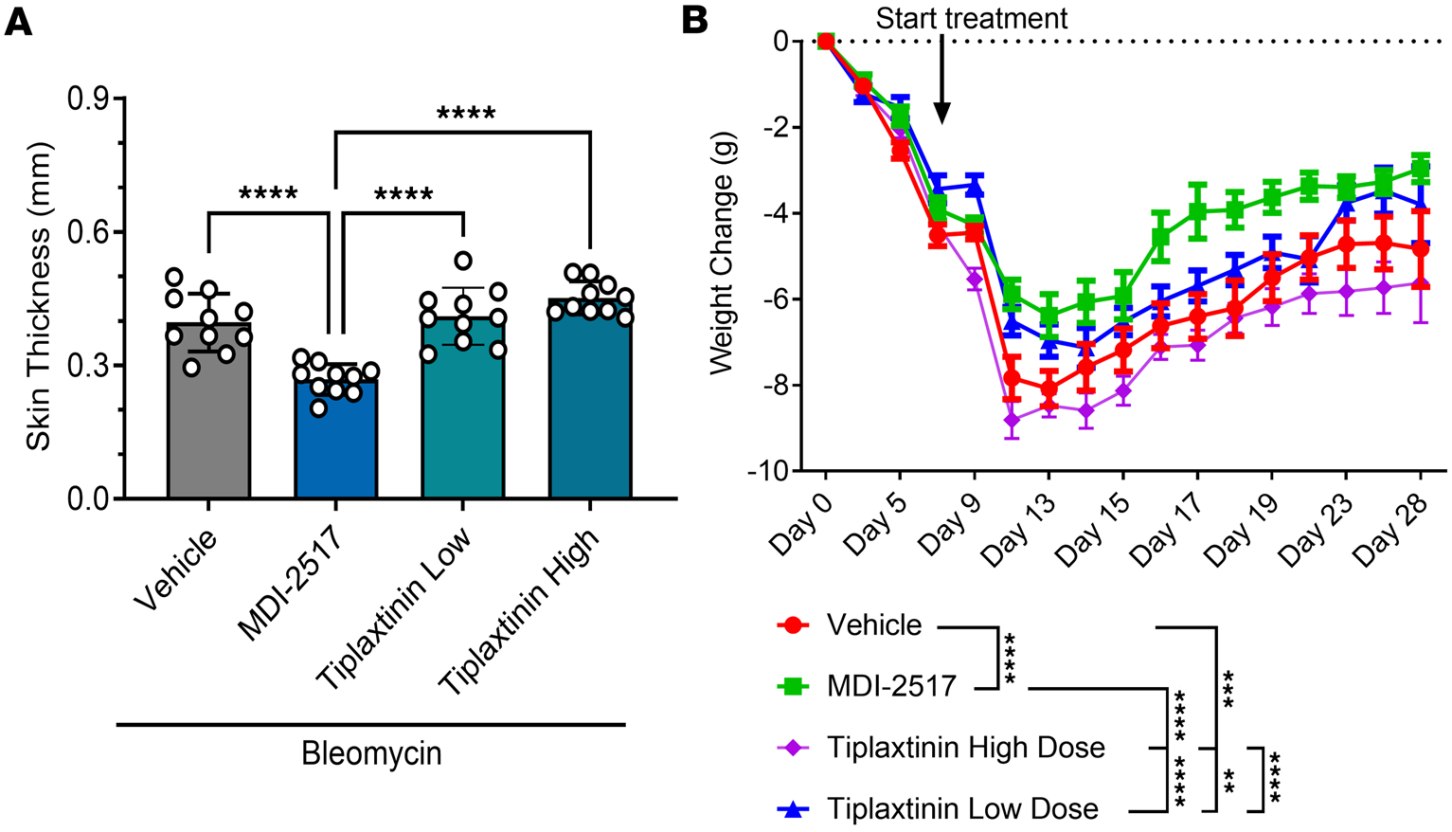
Comparator study of MDI-2517 and low-dose and high-dose tiplaxtinin. Twelve-week-old male C57BL/6J mice were subcutaneously implanted with osmotic pumps that delivered 100 U/kg (total) of bleomycin over 7 days. The pumps were then removed, and at that time the mice were placed on treatment chows (drug concentration in chow: vehicle, 0 mg/kg; MDI- 2517 at 500 mg/kg; low-dose tiplaxtinin at 500 mg/kg; or high-dose tiplaxtinin at 5,000 mg/kg). (**A**) On day 28, skin thickness was determined at multiple locations by skin pinch with calipers; then mice were sacrificed and tissues collected. (**B**) Body weight changes monitored approximately every 3 days. Data are shown as mean ± SD; *n* is indicated in each figure by the individual data points (*n* = 10); **P < 0.01, ***P < 0.001, ****P < 0.0001 by 1-way ANOVA in **A** and by a repeated-measures mixed-effects model in **B**.

**Figure 9:**
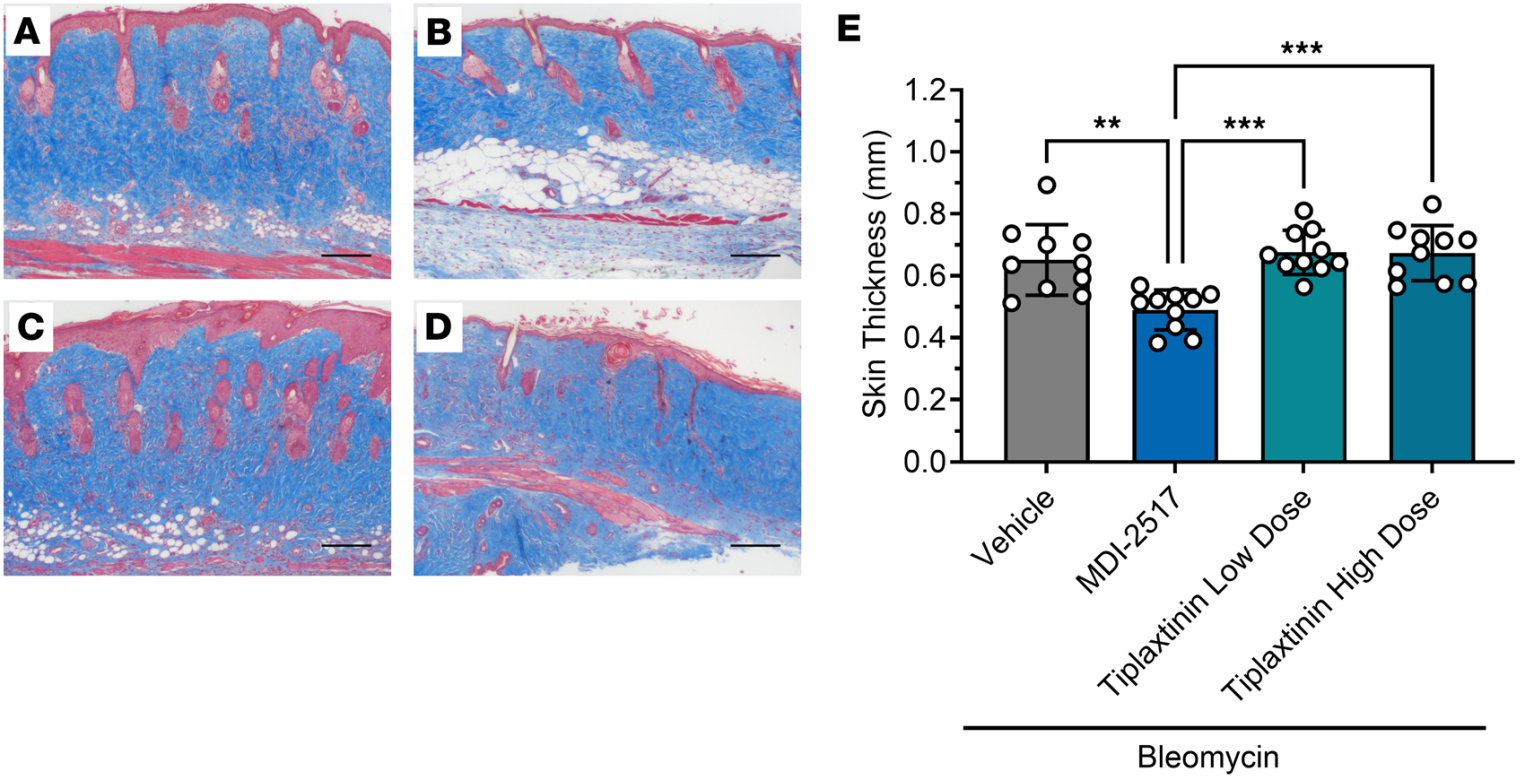
Skin thickness by Masson’s trichrome from comparator study of MDI-2517 and low-dose and high-dose tiplaxtinin. Twelve-week-old male C57BL/6J mice were subcutaneously implanted with osmotic pumps that delivered 100 U/kg (total) of bleomycin over 7 days. The pumps were then removed, and at that time the mice were placed on treatment chows (drug concentration in chow: vehicle, 0 mg/kg; MDI-2517 at 500 mg/kg; low-dose tiplaxtinin at 500 mg/kg; or high-dose tiplaxtinin at 5,000 mg/kg). On day 28, mice were sacrificed and skin tissues prepared for histological analysis by Masson’s trichrome stain. (**A**) Bleomycin control chow (no treatment). (**B**) Bleomycin MDI-2517 chow. (**C**) Bleomycin low-dose tiplaxtinin chow. (**D**) Bleomycin high-dose tiplaxtinin chow. (**E**) Quantification of skin thickness from Masson’s trichrome–stained slides. Scale bars: 100 μm. Data are shown as mean ± SD; *n* is indicated in each figure by the individual data points (*n* = 10); **P < 0.01, ***P < 0.001 by 1-way ANOVA.

We next compared the efficacy of MDI-2517 versus the PAI-1 inhibitor tiplaxtinin in our bleomycin SSc model. Tiplaxtinin has been studied in multiple animal models of diverse diseases (29, 30), including in a model of SSc (20). Two different doses of tiplaxtinin were tested: a low dose equivalent to the dose of MDI-2517 (500 mg/kg chow) and a high dose that has been shown to have efficacy in a murine model of atherosclerosis (5 g/kg chow) (31). MDI-2517 showed a significant reduction in skin thickness, while tiplaxtinin at either dose showed no reduction in comparison with vehicle controls (Figure 8A). Additionally, both MDI-2517–treated animals and animals treated with low-dose tiplaxtinin showed attenuation of weight loss, whereas the high-dose tiplaxtinin-treated cohorts had worse weight loss than control (Figure 8B). MDI-2517–treated mice also showed substantially less collagen deposition in the dermal layer (Figure 9B) in comparison with control or either dose of tiplaxtinin. Similar to Figure 6, the adipose layer also appeared well preserved in MDI-2517–treated samples (Figure 9B), and partially preserved with low-dose tiplaxtinin (Figure 9C), whereas this layer was largely absent in the vehicle controls (Figure 9A) as well as in the high-dose tiplaxtinin-treated cohort (Figure 9D). Quantification of skin thickness based on Masson’s trichrome staining (Figure 9E) showed protection against skin thickening with MDI-2517 treatment that was similar to that shown in Figure 8A, confirming the efficacy of MDI-2517 in reducing skin thickness using complementary skin pinch and Masson’s trichrome staining methods.

**Figure 10:**
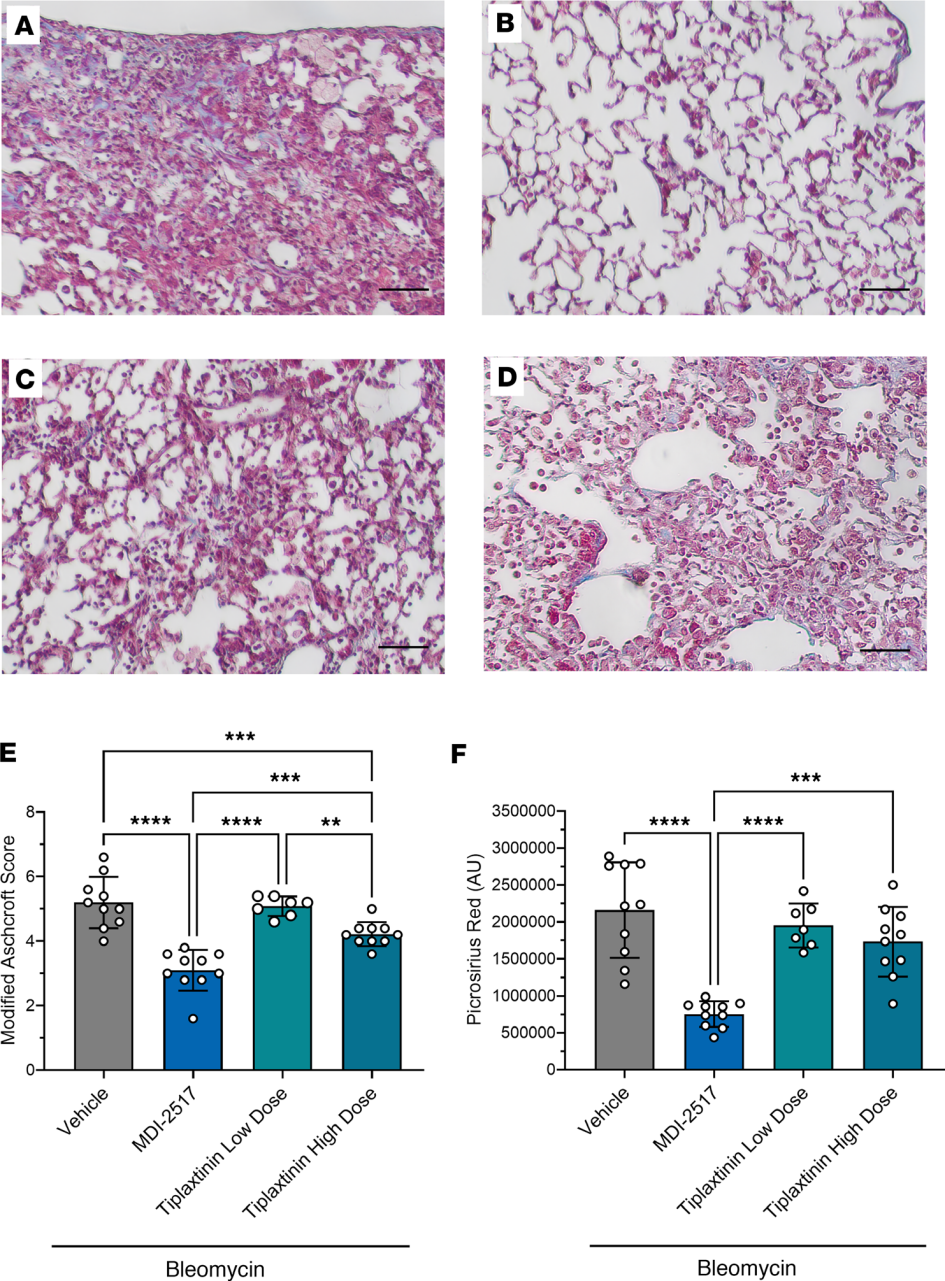
Lung trichrome staining from the comparator study of MDI-2517 and low-dose and high-dose tiplaxtinin. Twelve-week-old male C57BL/6J mice were subcutaneously implanted with osmotic pumps that delivered 100 U/kg (total) of bleomycin over 7 days. The pumps were then removed, and at that time the mice were placed on treatment chows (drug concentration in chow: vehicle, 0 mg/kg; MDI-2517 at 500 mg/kg; low-dose tiplaxtinin at 500 mg/kg; or high-dose tiplaxtinin at 5,000 mg/kg). On day 28, mice were sacrificed and lung tissues prepared for histological analysis by Masson’s trichrome stain. (**A**) Bleomycin control chow (no treatment). (**B**) Bleomycin MDI-2517 chow. (**C**) Bleomycin low-dose tiplaxtinin chow. (**D**) Bleomycin high-dose tiplaxtinin chow. (**E**) Modified Ashcroft score of lung tissue from Masson’s trichrome–stained slides. (**F**) Picrosirius red quantification from Masson’s trichrome–stained slides. Scale bars: 35 μm. Data are shown as mean ± SD; *n* is indicated in each figure by the individual data points (7 to 10); **P < 0.01, ***P < 0.001, ****P < 0.0001 in **E** and **F** by 1-way ANOVA.

Lung histology samples (Figure 10, A–D, and Supplemental Figure 7) were assessed by modified Ashcroft scoring for lung fibrosis, which demonstrated that both treatment with MDI-2517 and high-dose tiplaxtinin afforded protection. However, mice treated with MDI-2517 showed significantly lower scores (Figure 10E), suggesting that MDI-2517 is more efficacious in limiting injury-induced lung fibrosis than tiplaxtinin even at a 10-fold lower dose. This is consistent with previous studies of the closely related PAI-1 inhibitor MDI-2268, which demonstrated an approximately 10-fold higher potency compared with tiplaxtinin both in ex vivo plasma (22) and in vivo (31). MDI-2517 also showed a significant reduction in the amount of Picrosirius red staining (approximately 68% less than vehicle control). In contrast, tiplaxtinin showed a non-significant trend toward reduced Picrosirius red staining, but neither dose attained significance compared with the vehicle control (Figure 10F and Supplemental Figure 8).

## Discussion

SSc is a complex heterogeneous condition characterized by progressive fibrosis in multiple organs. Microvascular dysfunction is thought to be an early event in the development of SSc with vasculopathy and perivascular inflammation promoting fibroblast activation and myofibroblast differentiation (32–34). As vascular injury and perivascular inflammation are among the earliest pathological manifestations observed in SSc, it has been hypothesized that the development of tissue fibrosis in SSc might represent an exaggerated response to vascular damage, ischemia, and inflammation. Therefore, targeting factors with the potential to reduce vascular injury and inflammation has the potential to attenuate fibrotic development and could lead to new therapeutic approaches in SSc.

PAI-1 plays an important role regulating microvascular patency and is best understood for its role in regulating fibrinolysis by inhibiting the tissue-type and urokinase-type plasminogen activators (tPA and uPA), which convert the zymogen plasminogen to the active enzyme plasmin (35, 36). It has been shown that in patients with acute and chronic fibrotic diseases, there is a shift away from a net profibrinolytic activity due to a marked induction of PAI-1 (37–42). Recent data from single-cell gene expression studies in human lung transplant patients (43) and the data presented here in SSc skin (Figures 1 and 2) demonstrate that PAI-1 expression is prominently increased in these fibrotic disorders. PAI-1 expression is also increased in animal models of pulmonary fibrosis (12, 44–47) and SSc (21). More recently PAI-1 has also been shown to regulate inflammatory cell migration into sites of injury (12, 21, 48, 49). These observations indicate that PAI-1 is a key mediator of pathological vascular and inflammatory responses.

PAI-1 is also a primary regulator of wound healing (50, 51), including in the lung (52), consistent with a critical regulatory role in fibrosis. Wound healing is a natural repair process after injury that consists of overlapping stages, including hemostasis, inflammation, proliferation, matrix synthesis, and, finally, resolution. Disruption of this ordered process can result in impaired wound healing, leading to persistent inflammation and/or matrix synthesis and ultimately to a fibrotic syndrome (53). PAI-1, as a primary regulator of wound healing, has been shown to impact all stages of wound healing and, accordingly, to play a causal role in pulmonary fibrogenesis. Specifically, transgenic mice overproducing PAI-1 have been shown to accumulate excessive collagen following a fibrotic insult (54), whereas mice with a targeted PAI-1 gene deletion have been shown to be resistant to lung fibrosis and have improved survival following lung injury (54, 55). In addition, the suppression of PAI-1 through siRNA or by treatment with a pharmacological inhibitor limits lung scarring (19, 56). Mechanistically, excess PAI-1 promotes fibrosis by disrupting the ordered process of wound healing at several potential steps. First, by inhibiting fibrinolysis, PAI-1 supports the persistence of the proinflammatory provisional hemostatic fibrin matrix (16, 57). Second, PAI-1 enhances inflammatory cell infiltration through direct interaction with cellular integrins (21, 48, 49). This latter process may be particularly relevant to the development of pulmonary fibrosis, as PAI-1 has been shown to promote the recruitment of exudate macrophages to the lung (12) and to induce profibrotic M2 polarization in macrophages (58). PAI-1 has also been reported to directly promote myofibroblast differentiation and collagen synthesis (18) and to interact synergistically with TGF-β to sustain the fibrotic response (59-65). Very recently, PAI-1 has also been shown to promote lung fibrosis via a protease-independent mechanism through an interaction with sortilin-related receptor 1, a mosaic receptor involved in internalizing and intracellular trafficking of proteins (14, 66).

Multiple studies have implicated cellular senescence in the pathogenesis of SSc (67, 68), and our previous single-cell RNA-seq analysis of SSc skin found that the highest senescence scores were in fibroblasts, endothelial cells, and pericytes (69). A hallmark of senescent cells is the emergence of the senescence-associated secretory phenotype (SASP) (70). PAI-1 is a well-recognized SASP factor that has been linked to the regulation of cell senescence (65v, 71, 72) and aging (73, 74). The increased expression of PAI-1 in skin fibroblasts reported here is consistent with the high cellular senescence score observed in SSc skin fibroblasts (69), and suggests that PAI-1 may also exert its profibrotic influence through senescence-related mechanisms. Together these studies indicate that PAI-1 intersects with multiple pathways associated with the development of fibrotic disease and suggest that the inhibition of PAI-1 has the potential to impact the fibrotic response at multiple levels.

The present study demonstrates that the PAI-1 inhibitor MDI-2517 is efficacious in mitigating fibrotic changes in the bleomycin model of SSc. MDI-2517 treatment reduced fibrosis development in the skin in a dose-dependent manner and attenuated weight loss associated with the bleomycin model. Consistent with prior reports that dermal adipocytes play a key role in the pathogenesis of cutaneous fibrosis (75), our histological analyses also revealed that MDI-2517 treatment, in addition to reducing dermal thickening and collagen deposition, also preserved the intradermal adipose layer. Loss of this adipose compartment is a hallmark of fibrotic remodeling, and previous studies have demonstrated that adiponectin-positive adipocyte progenitors can transdifferentiate into myofibroblasts, thereby contributing to fibrosis development (76). Moreover, adipocytes secrete antifibrotic cytokines that may help limit excessive ECM deposition (77). Thus, the preservation of dermal adipose tissue in MDI-2517–treated animals may underlie, at least in part, the compound’s protective effects against bleomycin-induced dermal fibrosis.

Common biomarkers of fibrosis were also reduced by MDI-2517 treatment. Notably, MDI-2517 demonstrates better efficacy than other drugs, such as pirfenidone, MMF, or tiplaxtinin. Further, MDI-2517 demonstrated antifibrotic activity in SSc-related lung fibrosis. The first-line standard of care for SSc, MMF, has been shown to slow disease progression; however, it does not stop or reverse progression. Thus, it is imperative that more effective therapeutics be developed. MDI-2517 would be a first-in-class, orally administered once daily, small-molecule inhibitor of PAI-1. Our preclinical data suggest that MDI-2517 may have the potential to halt fibrosis progression, which, if demonstrated in humans, would constitute a substantial advancement over the current standard of care for patients with SSc.

The precise mechanism whereby MDI-2517 reduces fibrogenic progression, collagen accumulation, and disease severity is not known. As discussed above, PAI-1 intersects multiple pathways associated with fibrotic disease, including fibrinolysis, inflammation, senescence, and wound healing; MDI-2517 inhibition of PAI-1 could impact all of these processes. However, the data in Figure 2 demonstrating the ability of MDI-2517 to significantly downregulate *COL1A1* and *ACTA2* expression suggest that a major effect of MDI-2517 may be on the myofibroblast phenotype via inhibition of a PAI-1–mediated signaling pathway that promotes fibrogenic gene expression. It is also important to note that, although PAI-1 plays an important role in wound healing, inhibition of PAI-1 with MDI-2517 at the dose used in this study did not appear to impair tissue repair, as no delays in skin wound closure were observed following osmotic pump removal (data not shown). We believe that this is consistent with the reductions in PAI-1 skin and plasma seen in Figure 3 where MDI-2517 treatment lowers PAI-1 closer to the normal range without producing a state resembling PAI-1 deficiency.

A limitation of our study is that only male mice were used. In humans SSc is more common in women than in men; however, men often experience a more severe disease course (78). In our studies we chose to use male mice because of their reported enhanced sensitivity to bleomycin-induced fibrosis compared with females (79, 80). However, in future studies we will compare the response to MDI-2517 treatment in male and female mice to determine whether the benefit seen in male mice is also apparent in females. Another limitation is the lack of pharmacokinetic data due to the administration of drugs via chow, and additional studies with gavage and intravenous dosing will be necessary in the future.

In summary, MDI-2517 has demonstrated remarkable effectiveness in our preclinical model of SSc. However, further studies are warranted to understand the underlying mechanisms by which it exerts its beneficial effects. It will also be important to explore and confirm its efficacy in other preclinical models of fibrosis representative of different organ systems and etiologies. Such studies will provide further understanding of the specific pathways and molecular targets engaged by PAI-1 that are inhibited by MDI-2517 treatment and provide valuable insights into its potential applicability in different organ-specific fibrotic conditions. While the development of targeted therapies for SSc, including those directed at PAI-1, is still in its early stages, these advancements hold promise for the future of SSc treatment. Further research and clinical trials are needed to establish the safety and efficacy of these therapies in future studies.

## Methods

### Sex as a biological variable.

The human samples were from both male and female patients and controls (Supplemental Table 2). Analysis of skin fibroblasts from both male and female donors demonstrated no significant difference in *SERPINE1* expression in either patient or healthy control cells (*P* = 0.668 and *P* = 0.796, respectively). For the mouse experiments, only males were used because of their reported enhanced sensitivity to bleomycin-induced fibrosis compared with females (79, 80), and therefore sex was not considered as a biological variable.

### Patients and controls.

Study participants were recruited from the University of Michigan Scleroderma Program. Dermal fibroblasts were isolated from punch biopsies from the distal forearm of healthy volunteers and diffuse cutaneous SSc patients. All patients met the American College of Rheumatology/ European Alliance of Associations for Rheumatology criteria for the classification of SSc (81). As summarized in Supplemental Table 2, all patients were diagnosed with diffuse cutaneous SSc (6 males and 20 females; age 54.0 ± 2.7 years, mean ± SEM), and the disease duration was 2.7 ± 0.5 years (mean ± SEM). Their skin scores ranged from 0 to 40 with a mean of 16.3 ± 2.1 (mean ± SEM). Twenty-five patients had Raynaud’s phenomenon, 5 had pulmonary arterial hypertension, and 13 had interstitial lung disease at the time of biopsy. Twenty-five patients were on immunosuppressant therapy. Age-, sex-, and ethnicity-matched healthy controls were also recruited (age 52.7 ± 4.4 years, mean ± SEM; 4 males and 12 females). Separately, 22 SSc patients and 18 healthy controls were recruited for single-cell RNA-seq, and an additional four SSc patients were recruited for spatial sequencing (23). Skin biopsies were taken from the affected forearms of patients. Both studies were approved by the University of Michigan Institutional Review Board, and all patients gave written consent. The studies were conducted according to the Declaration of Helsinki principles.

### Single-cell RNA-seq library preparation, sequencing, and alignment.

Single-cell RNA-seq library preparation, sequencing, and alignment were performed as previously described by our group (82). After processing, libraries were then sequenced on the Illumina NovaSeq 6000 sequencer to generate 150 bp paired-end reads. Data processing, including quality control, read alignment (hg38), and gene quantification, was conducted using 10x Genomics Cell Ranger software. The samples were then merged into a single expression matrix using the cellranger aggr pipeline. The R package Seurat (v3.1.2) was used to cluster the cells in the merged matrix. Cells with less than 500 transcripts or 100 genes or more than 1 × 10^5^ transcripts or 15% of mitochondrial expression were first filtered out as low-quality cells. Subclustering was performed on the abundant cell types. The same functions described above were used to obtain the subclusters. Subclusters that were defined exclusively by mitochondrial gene expression, indicating low quality, were removed from further analysis.

### Spatial sequencing library preparation.

Skin samples were frozen in OCT medium and stored at –80°C until sectioning. Optimization of tissue permeabilization was performed on 20 μm sections using a Visium Spatial Tissue Optimization Reagents Kit (10x Genomics), which established an optimal permeabilization time of 9 minutes. Samples were mounted onto a Gene Expression slide (10x Genomics). Libraries were then sequenced on the Illumina NovaSeq 6000 sequencer to generate 150 bp paired-end reads. After sequencing, the reads were aligned to the human genome (hg38), and the expression matrix was extracted using the Space Ranger pipeline. Seurat was then used to analyze the expression matrix. Specifically, the SCTransform function was used to scale the data and find variable genes with default parameters. PCA and uniform manifold approximation and projection (UMAP) were applied for dimensional reduction. The FindTransferAnchors function was used to find a set of anchors between the spatial sequencing data and single-cell RNA-seq data, which were then transferred from the single-cell RNA-seq to the spatial sequencing data using the TransferData function.

### Immunohistochemistry staining.

Paraffin-embedded tissue sections from human skin (SSc and normal) were heated at 60°C for 30 minutes, deparaffinized, rehydrated, and counterstained with hematoxylin. Slides were placed in PH9 antigen retrieval buffer and heated at 125°C for 30 seconds in a pressure cooker. After cooling, slides were treated with 3% H_2_O_2_ for 5 minutes and then blocked using 10% goat serum for 30 minutes. Overnight incubation (4°C) was then performed using anti–human PAI-1 (Innovative Research, ASHPAI-GF-HT) at a dilution of 1:300. Slides were then washed, treated with peroxidase-labeled secondary antibody (Abcam, ab6802) for 30 minutes, and reacted with the diaminobenzidine substrate.

### Cell culture.

Punch biopsies obtained from healthy subjects and SSc patients were digested as previously described (83, 84). Dermal fibroblasts were maintained in RPMI supplemented with 10% fetal bovine serum, l-glutamine, and antibiotics. Cells between passages 3 and 6 were used in all experiments.

### Cell treatment.

Dermal fibroblasts from diffuse cutaneous SSc patients were treated with 100 μM of MDI-2517 or vehicle for 48 hours before quantitative reverse transcriptase PCR measurement for *ACTA2* and *COL1A1* and for 72 hours before immunoblotting of αSMA and COL1.

### mRNA extraction and quantitative reverse transcriptase PCR.

Total RNA was extracted using Direct-zol RNA MiniPrep Kit (Zymo Research) before being converted to cDNA using the Verso cDNA synthesis kit (Thermo Fisher Scientific). Quantitative PCR was performed using SYBR Green PCR Master Mix (Applied Biosystems) with specific primers for *COL1A1*, *ACTA2*, and *SERPINE1*, with *ACTB* as reference gene for normalization. We assessed *ACTB* expression levels across the SSc fibroblasts and healthy controls and found no significant differences, confirming its suitability as a reference gene (KiCqStart SYBR Green Primers, MilliporeSigma). All samples were run in duplicate using the ViiA 7 Real-Time PCR System. Data were analyzed using the Applied Biosystems software.

### Western blots.

Western blotting was performed following a protocol described previously (84). Briefly, equal amounts of cell lysates were loaded onto polyacrylamide gels and separated by SDS-PAGE. The proteins were then transferred onto nitrocellulose membranes via Western blotting. After blocking, the blots were probed with antibodies against collagen I (Abcam, ab6308), PAI-1 (Innovative Research, IRBAHUPAI1AP100UG), or αSMA (Abcam, ab5694). For loading control, the blots were immunoblotted with antibodies against GAPDH (Cell Signaling, 2118) or vinculin (Sigma-Aldrich, V9131). Band quantification was performed using ImageJ (85) or with Image Studio (LICORbio).

### Murine model of SSc.

All animal procedures were performed in accordance with the local welfare legislation and approved by the Institutional Animal Care and Use Committee at the University of Michigan. Twelve-week-old male C57BL/6 mice (The Jackson Laboratory) received bleomycin via osmotic minipumps (ALZET 1007D, DURECT). The minipumps were loaded with bleomycin (100 U/kg). Mice were anesthetized with isoflurane, and the pumps were implanted subcutaneously under the loose skin on the back of the mice slightly posterior to the scapulae. Pumps were removed on day 7 as recommended by the manufacturer. The incision was closed with a wound clip. After pump removal, animals were placed on synthetic laboratory chow (Dyets Inc.) supplemented with the PAI-1 inhibitor MDI-2517 (MDI Therapeutics Inc.) at various doses (250 mg/kg, 500 mg/kg, and 1,000 mg/kg), pirfenidone (1,000 mg/kg; Cipla), mycophenolate (1,000 mg/kg; Accord Health), high-dose (5,000 mg/kg) tiplaxtinin (synthesized by Scott D. Larsen, University of Michigan College of Pharmacy, as previously described; ref. 86), or low-dose (500 mg/kg) tiplaxtinin, or control chow without any drugs. The amount of chow consumed for each formulation was monitored by cages to ensure that there were no significant differences between groups. For all studies presented except the pilot study in Supplemental Figure 3, all mice receiving bleomycin were given daily subcutaneous saline injections (10 mL/kg) from day 9 to day 16 to prevent dehydration. This reduced overall mortality from 20% (4 of 20) in the pilot study to 2.7% (3 of 110) in all other studies. Control groups not treated with bleomycin were not given saline injections.

### Tissue collection.

Four weeks after pump implantation, mice were anesthetized and transcardially perfused with PBS, and then the trachea was cannulated, and the lung tissues were inflated and fixed with 10% neutral phosphate-buffered formalin in situ. The fixed lungs were processed and embedded in paraffin for microscopy. Skin samples were collected at the region of pump delivery as well as an area approximately 5 cm away and processed for histology.

### Morphometric analysis.

For histopathological evaluation of fibrosis, mouse lungs and skin were fixed in 10% neutral phosphate-buffered formalin. The paraffin-embedded tissues were cut into 5 μm sections and stained with Masson’s trichrome. Fibrosis was assessed using a modified Ashcroft score for lung tissues, with a numerical fibrosis scoring scale (0 to 8), in Masson’s trichrome–stained sections (87). Scoring was performed by a blinded investigator. Dermal thickness was measured in Masson’s trichrome–stained sections by measurement of the distance between the epidermal-dermal junction and the dermal-fat junction in 5 randomly selected fields in 2 or more sections from each animal. The data shown are from the skin samples collected proximal to pump delivery.

### Picrosirius red staining and quantification.

Deparaffinization sections (5 μm) were immersed in Picrosirius red solution (0.1% wt/vol Direct Red 80; Sigma-Aldrich, 365548) in a saturated aqueous solution of picric acid (Sigma-Aldrich) for 1 hour. Sections were briefly rinsed in 2 changes of acidified dH_2_O (0.5% glacial acetic acid), dehydrated, cleared, and mounted. Digital images were obtained with a Nikon Microphot-SA microscope and a Nikon DS-Fi3 camera using NIS-Elements software (Nikon Inc.) and analyzed using ImageJ software. Microscope conditions (lamp brightness, condenser opening, objective, zoom, exposure time, and gain parameters) were maintained throughout the imaging of all samples. Collagen content was quantified using signal threshold settings.

### PAI-1 and biomarkers quantification.

Selected biomarkers including surfactant protein-D (SP-D), intercellular adhesion molecule 1 (ICAM-1), and MMP-9 were quantified in plasma samples from mice drawn on day 28 at the time the mice were sacrificed, and biomarkers were measured on a Luminex X-100 (Luminex Corp.) using a 3-plex Luminex panel (R&D Systems, LXSAMSM-03). The concentration of total and active murine PAI-1 in plasma was measured using a magnetic carboxylated microsphere–based ELISA as previously described (14).

### Immunofluorescence.

Quantification of PAI-1 immunofluorescence in mouse skin tissue was obtained from 5-μm-thick paraffin sections. Slides were dried on a slide warmer at 55°C. Slides were deparaffinized with Histo-Clear (Fisher Scientific), rehydrated by incubation through a graded series of ethanol from 100% to 50%, and rinsed with tap water. Slides were then rinsed in PBS and subjected to antigen retrieval (Dako, S1700), then permeabilized in PBS plus 0.5% Triton X-100 (PBST), after which they were blocked overnight with PBST plus 5% bovine serum albumin. Sections were next incubated with primary antibody (monoclonal H34G6, Innovative Research) in PBST with 1% horse serum for 2 hours at room temperature, washed 3 times with PBST, and then developed by incubation with an Alexa Fluor 568 secondary antibody for 1 hour at room temperature. Sections were washed 3 times with PBST and incubated in PBST plus DAPI for a final 10-minute incubation at room temperature. Sections were washed 3 times with PBST and a final wash in PBS alone. Coverslips were added to slides with VECTASHIELD HardSet Mounting Medium (Vector Laboratories, H1400-10). Digital images were captured by a Nikon Microphot-SA microscope and PCO Panda camera (Nikon Inc.) with NIS-Elements software and analyzed using ImageJ software. PAI-1 levels were quantified using signal threshold settings.

### Statistics.

Data analysis was performed using GraphPad Prism 8 statistical software (GraphPad Software). For in vivo experiments, *n* indicates the number of individual mice used in the study. For statistical analysis, in any experiment with only 2 groups, a 2-tailed *t* test or Mann-Whitney test was used. For experiments with more than 2 groups, a 1-way ANOVA with appropriate post hoc test for multiple comparisons was used, except for qPCR comparisons, where a 1-sample Wilcoxon’s test was used. For analysis of weight loss, the data were analyzed by a repeated-measures mixed-effects model with an appropriate post hoc test for multiple comparisons. In all graphs the relevant statistical comparisons are shown, and all statistical comparisons are given in Supplemental Table 3. Data are represented as mean values ± SD; *P* < 0.05 was considered significant.

### Study approval.

The study was approved by the University of Michigan Institutional Review Board, and all patients gave written informed consent. The study was conducted according to the Declaration of Helsinki principles. All animals were housed in the University of Michigan animal facility, provided with food and water ad libitum, and all procedures were conducted under approved University of Michigan Institutional Animal Care and Use Committee protocols.

### Data availability.

The RNA-seq data can be accessed on GEO (GSE249279). All data from this study are accessible in the supplemental material, the Supporting Data Values file, and public repositories.

## Author contributions

EJS and PST designed and performed experiments, analyzed data, and wrote the manuscript. MW, NS, KM, SV, AR, LL, XX, EX, OP, RB, and LCT performed experiments and acquired data. CDE provided reagents and edited the manuscript. DK, JV, and THS provided advice on experiments and edited the manuscript. JEG and DAL designed experiments, analyzed data, and wrote the manuscript. All authors read and approved the final manuscript.

## Funding support

This work is the result of NIH funding, in whole or in part, and is subject to the NIH Public Access Policy. Through acceptance of this federal funding, the NIH has been given a right to make the work publicly available in PubMed Central.

- National Heart, Lung, and Blood Institute of the NIH awards R01HL055374 and R01HL163870 (to DAL).
- National Institute on Aging of the NIH award R01AG074552 (to DAL).
- National Institute of Arthritis and Musculoskeletal and Skin Diseases of the NIH award P30AR075043 (to JEG).
- National Institute of Allergy and Infectious Diseases of the NIH award RO1AI183620 (to JEG).
- National Institute of Arthritis and Musculoskeletal and Skin Diseases of the NIH awards R43AR074318 and R44AR074318 to MDI Therapeutics, with subcontracts to DAL.
- National Heart, Lung, and Blood Institute of the NIH awards R43HL145960 and R44HL158435 to MDI Therapeutics, with subcontracts to DAL.

## Supplementary Material

Supplemental data

Supporting data values

## Version 1 02/17/2026

In-Press Preview

## Version 2 04/08/2026

Electronic publication
