# Supplementary material for: A potent inhibitor of PAI-1, MDI-2517, mitigates disease severity in a preclinical systemic sclerosis model
Source: JCI Insight. 2026 Feb 17;11(7):e195005. doi: 10.1172/jci.insight.195005 (PMC13134730; doi:10.1172/jci.insight.195005)
Supplement: Supplemental data [file jciinsight-11-195005-s075.pdf]

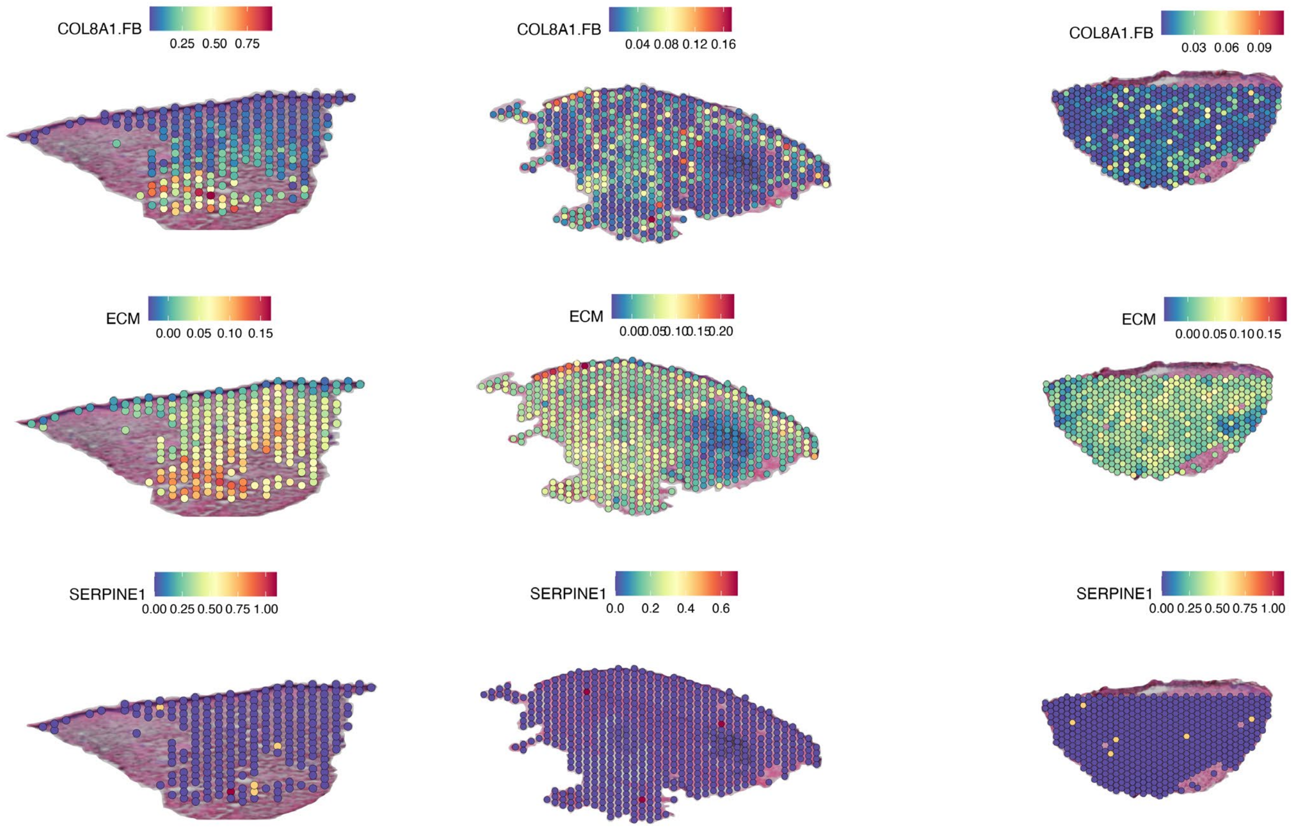

Figure S1. Visium spatial sequencing analyses. Visium data for COL8A1, SERPINE1 and ECM module.

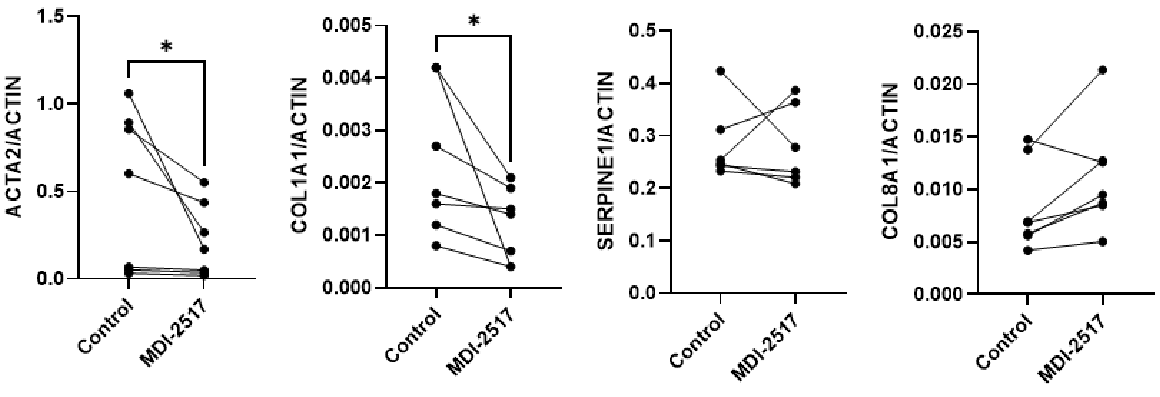

Figure S2. Downregulate pro-fibrotic markers in human SSc fibroblasts by MDI-2517. The non-normalized data from Figure 2B. Dermal fibroblasts from SSc patients were treated with MDI-2517 and gene expression of various genes were quantified by qPCR.* p<0.05 by two-tailed t-test.

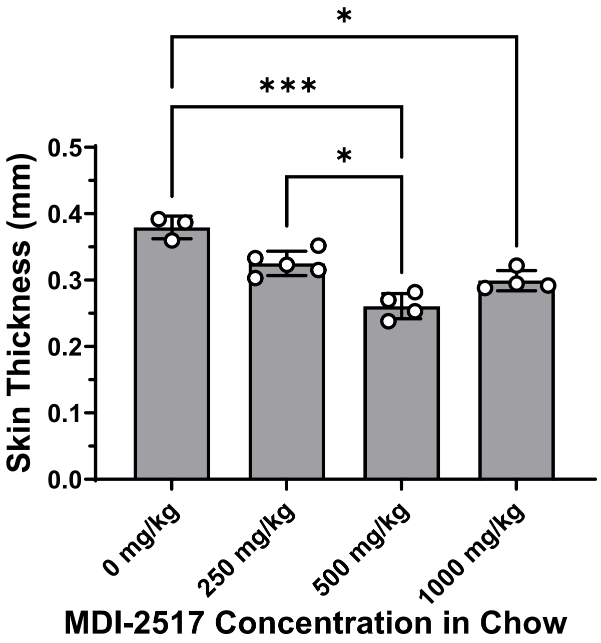

Figure S3. Dose response of MDI-2517 in bleomycin scleroderma model: Twelve-week-old male C57BL/6J mice were subcutaneously implanted with osmotic pumps that delivers 100U/kg (total) of bleomycin over 7 days or saline. The pumps were then removed and at that time the mice were placed on either control chow (0 mg/kg) or chow containing MDI-2517 at concentrations of drug in chow of 250mg/kg, 500mg/kg, or 1000mg/kg. On day 28 skin thickness was determined at multiple location by skin pinch with calipers. Data is shown as mean ± SD, n is indicated in each figure by the individual data points (3-5), * $p<0.05$, *** $p<0.001$, by Kruskal-Walls test.

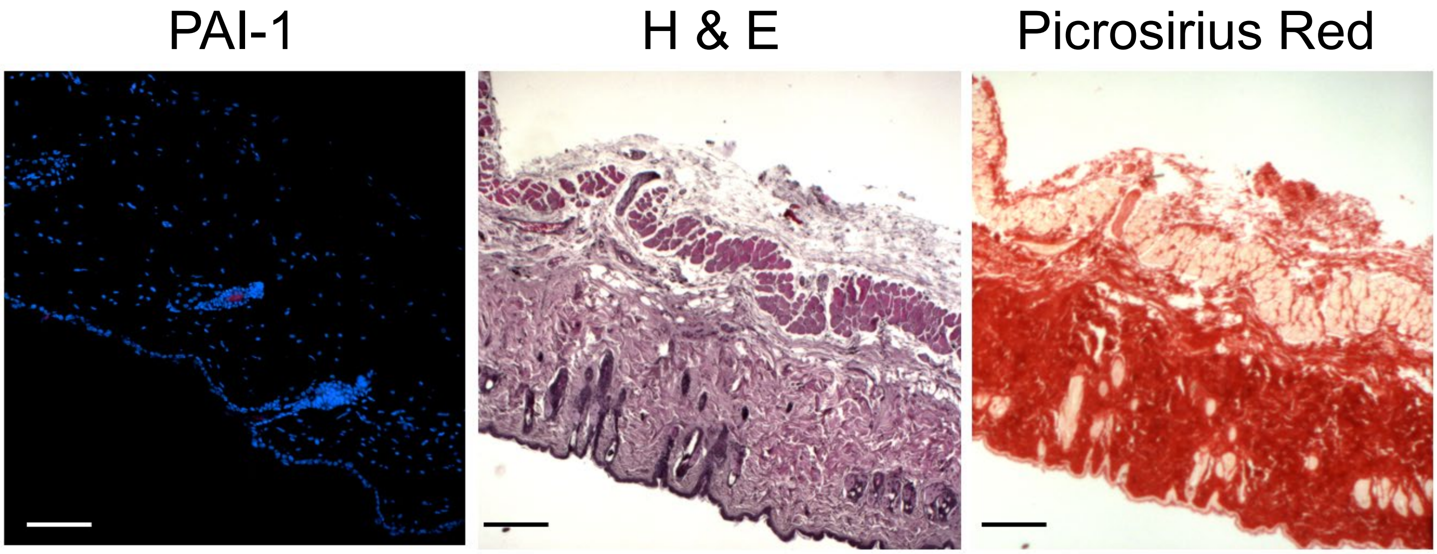

Figure S4. Naïve mouse skin. Skin from naïve twelve-week-old male C57BL/6J mice. A) PAI-1 antigen in skin (red) and DAPI nuclear stain (blue), B) hematoxylin and eosin (H&E) C) Picrosirius Red staining, scale bar = 100μm.

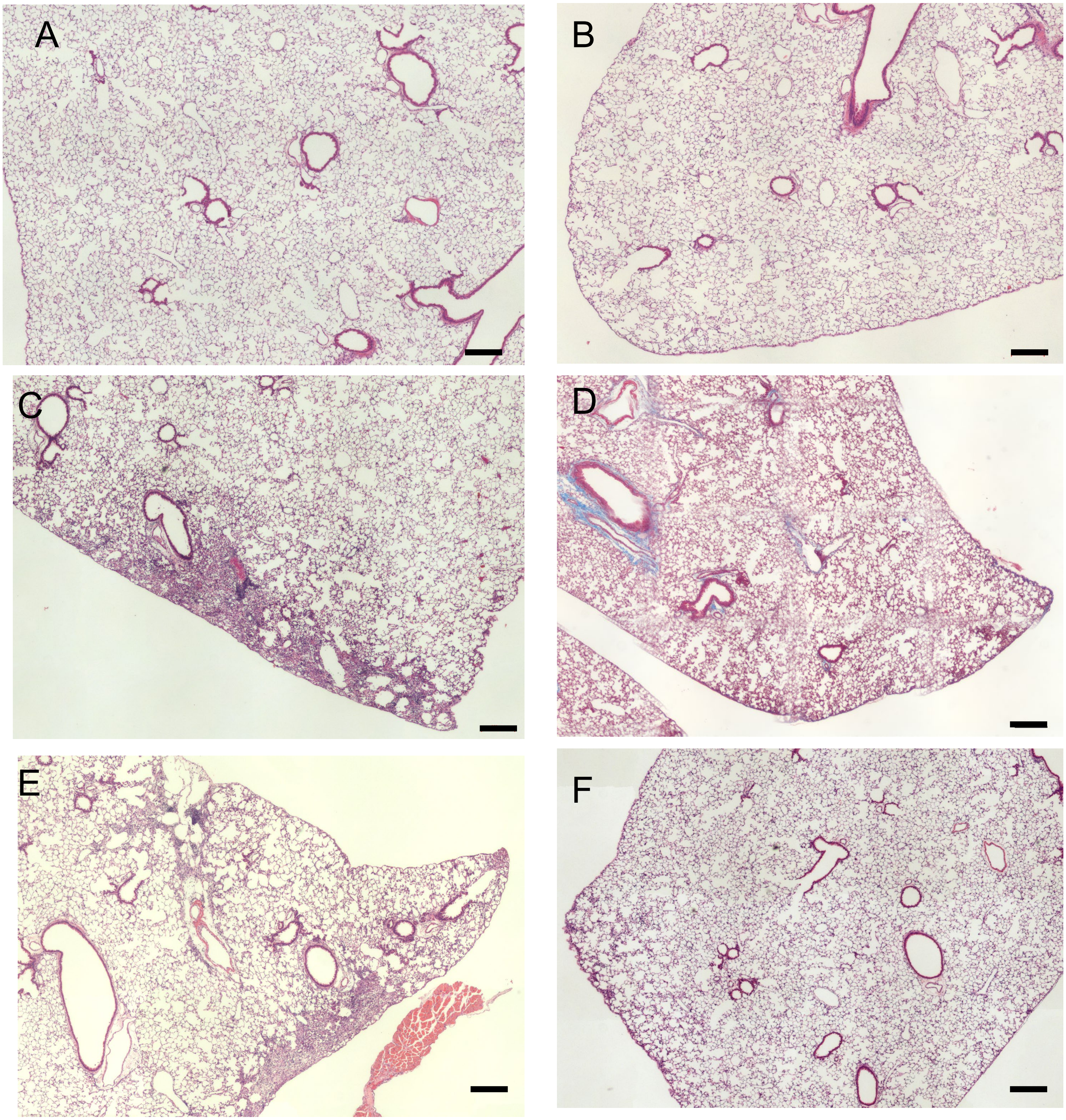

Figure S5, Low Resolution Images of Lung trichrome staining from the comparator study of MDI-2517, mycophenolate (MMF) and combined MMF+MDI-2517. Twelve-week-old male C57BL/6J mice were subcutaneously implanted with osmotic pumps that delivers 100U/kg (total) of bleomycin or saline over 7 days. The pumps were then removed, and at that time the mice were placed on treatment chows (drug concentration in chow: Vehicle, 0 mg/kg, MDI-2517 at 500 mg/kg, MMF at 1000mg/kg or combined MDI-2517 at 500 mg/kg and MMF at 1000 mg/kg,). On day 28, lung tissues were prepared for histological analysis by Mason Trichrome Stain. A) Saline Control Chow, B) Saline MDI-2517 chow, C) Bleomycin control chow (No Treatment), D) Bleomycin MDI-2517 chow, E) Bleomycin MMF chow, F) Bleomycin combined MMF and MDI-2517 chow, Scale Bar = 250 μm

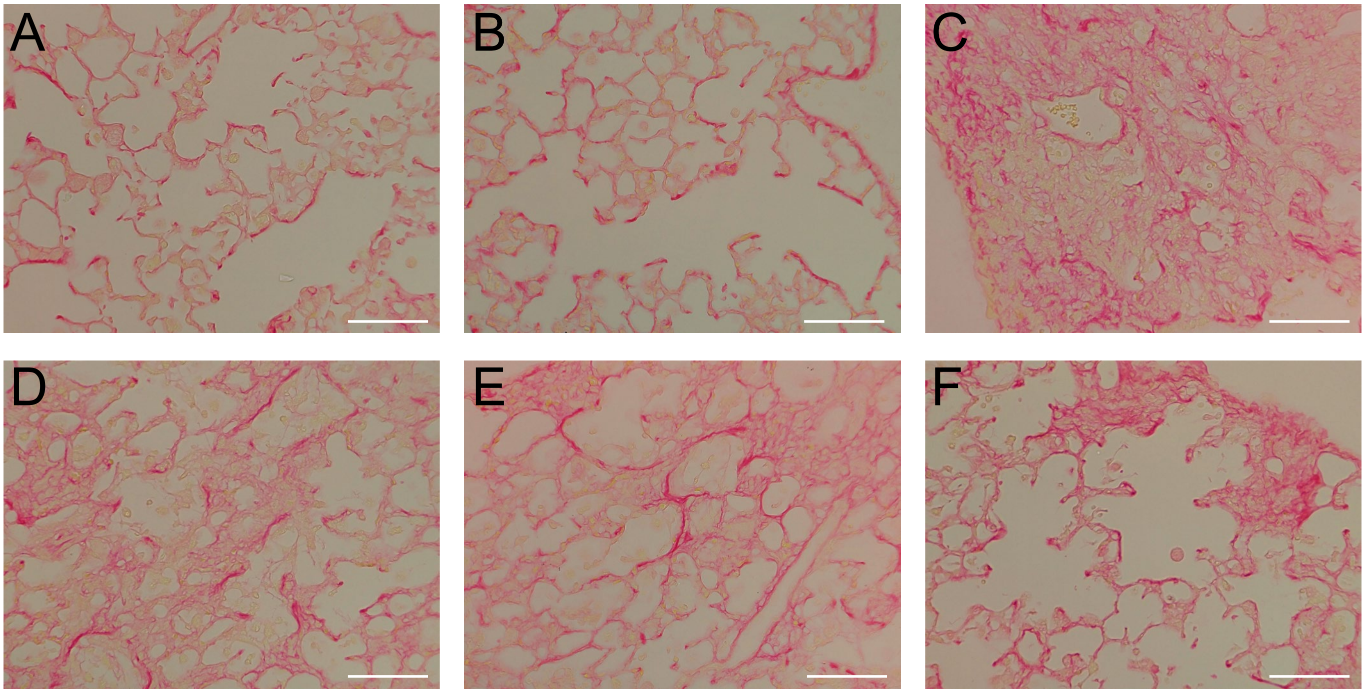

Figure S6, Lung Picrosirius Red staining from the comparator study of MDI-2517, mycophenolate (MMF) and combined MMF+MDI-2517. Twelve-week-old male C57BL/6J mice were subcutaneously implanted with osmotic pumps that delivers 100U/kg (total) of bleomycin or saline over 7 days. The pumps were then removed, and at that time the mice were placed on treatment chows (drug concentration in chow: Vehicle, 0 mg/kg, MDI-2517 at 500 mg/kg, MMF at 1000mg/kg or combined MDI-2517 at 500 mg/kg and MMF at 1000 mg/kg,). On day 28, lung tissues were prepared for histological analysis. A) Saline Control Chow, B) Saline MDI-2517 chow, C) Bleomycin control chow (No Treatment), D) Bleomycin MDI-2517 chow, E) Bleomycin MMF chow, F) Bleomycin combined MMF and MDI-2517 chow. Scale bar = 50µm.

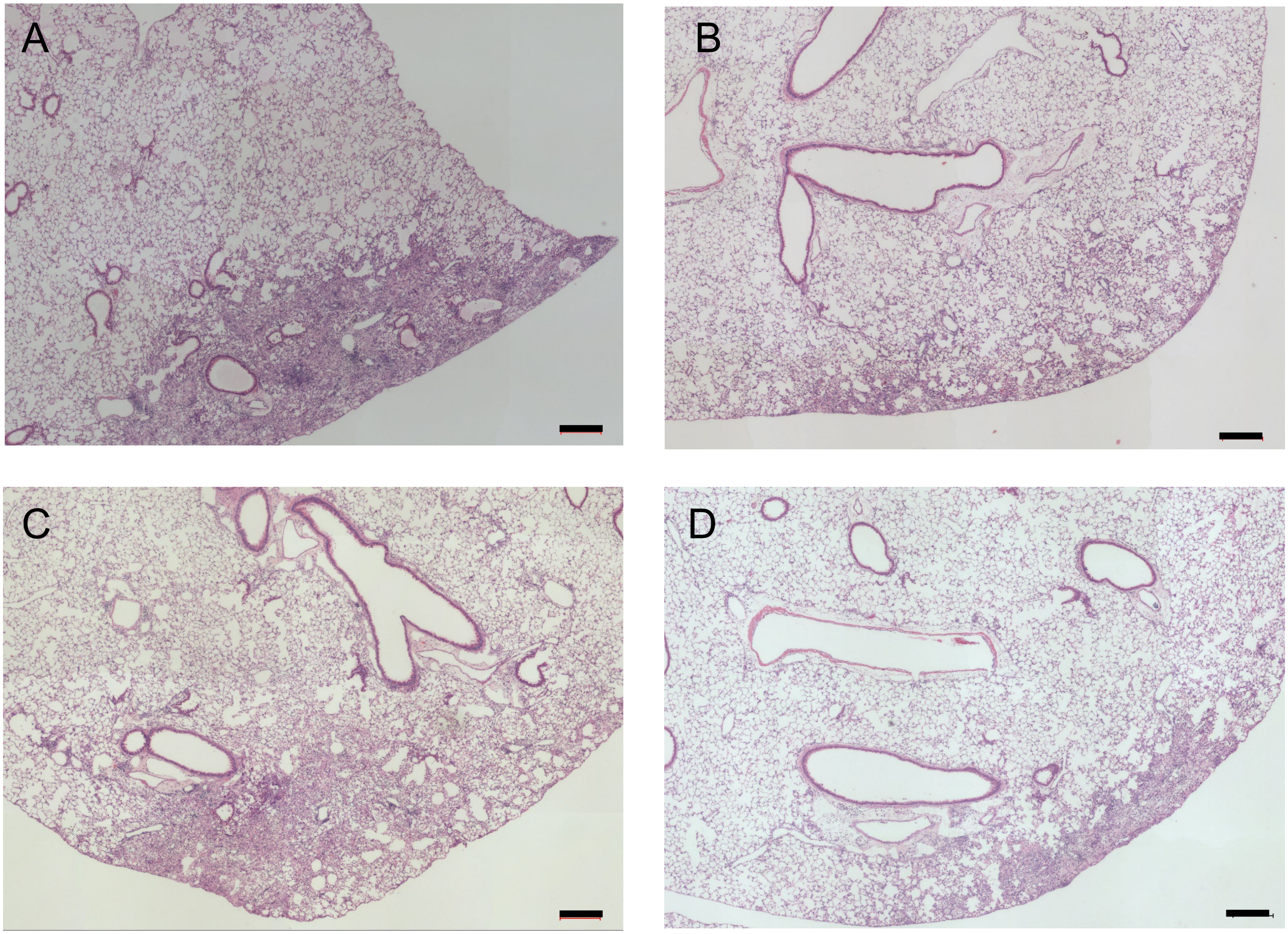

Figure S7, Low Resolution Images of Lung trichrome staining from the comparator Study MDI-2517, Tiplaxtinin Low and High Dose. Twelve-week-old male C57BL/6J mice were subcutaneously implanted with osmotic pumps that delivers 100U/kg (total) of bleomycin over 7 days. The pumps were then removed, and at that time the mice were placed on treatment chows (drug concentration in chow: Vehicle, 0 mg/kg, MDI-2517 at 500 mg/kg, Tiplaxtinin Low dose at 500 mg/kg or Tiplaxtinin High dose at 5000 mg/kg). On day 28 mice were sacrificed and lung tissues prepared for histological analysis by Mason Trichrome stain. A) Bleomycin control chow (No Treatment), B) Bleomycin MDI-2517 chow, C) Bleomycin Tiplaxtinin Low dose chow, D) Bleomycin Tiplaxtinin High dose chow. Scale Bar = 250 μm

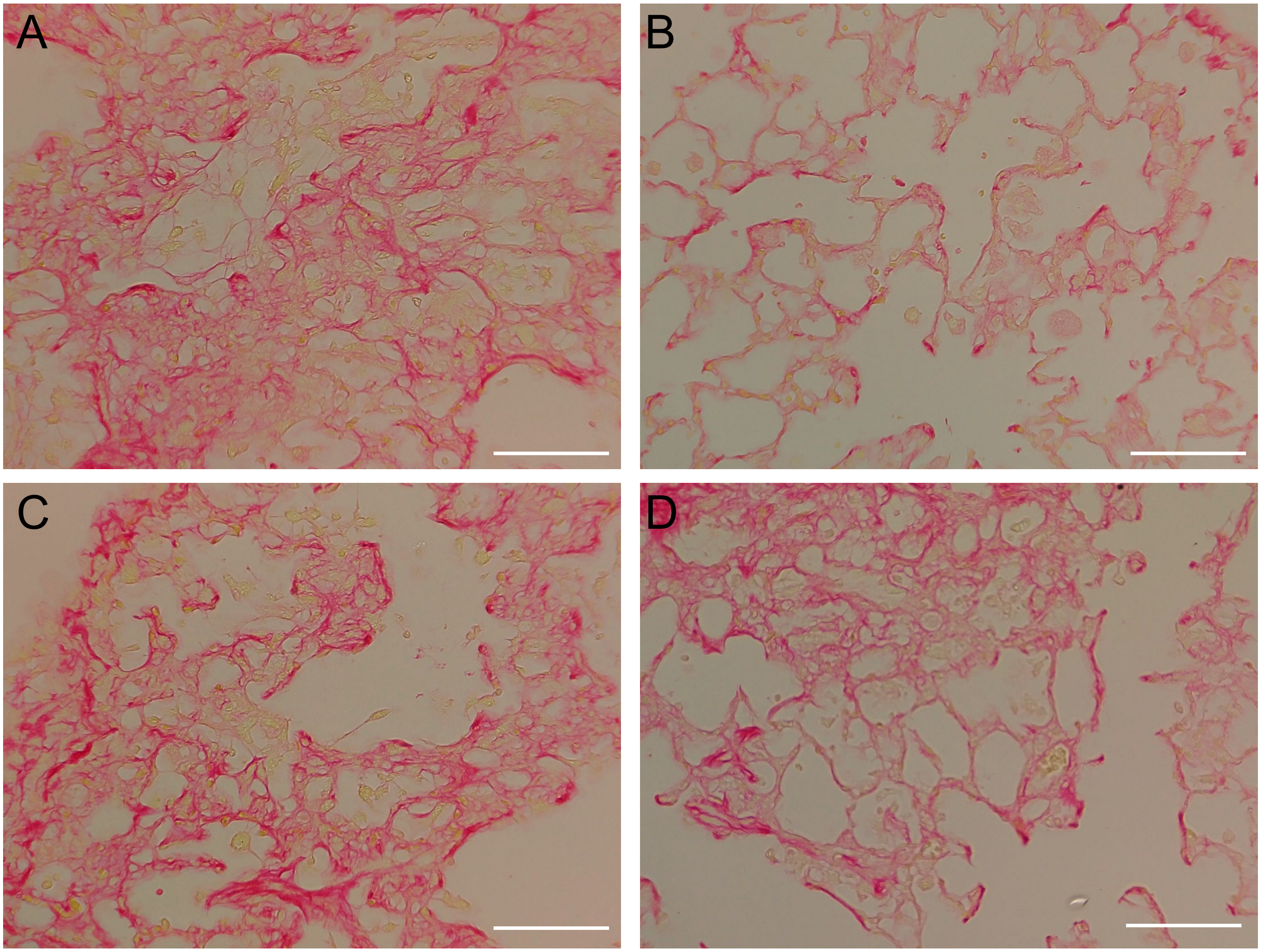

Figure S8, Lung Picrosirius Red staining from the comparator Study MDI-2517, Tiplaxtinin Low and High Dose. Twelve-week-old male C57BL/6J mice were subcutaneously implanted with osmotic pumps that delivers 100U/kg (total) of bleomycin over 7 days. The pumps were then removed, and at that time the mice were placed on treatment chows (drug concentration in chow: Vehicle, 0 mg/kg, MDI-2517 at 500 mg/kg, Tiplaxtinin Low dose at 500 mg/kg or Tiplaxtinin High dose at 5000 mg/kg). On day 28 mice were sacrificed and lung tissues prepared for histological analysis. A) Bleomycin control chow (No Treatment), B) Bleomycin MDI-2517 chow, C) Bleomycin Tiplaxtinin Low dose chow, D) Bleomycin Tiplaxtinin High dose chow. Scale bar = 50µm.

Supplementary Table S1: Gene components of our ECM module score

Symbol	Symbol	Symbol	Symbol	Symbol	Symbol	Symbol
A2M	COL11A2	COLGALT1	IBSP	LAMB1	MMP8	TIMP1
ACAN	COL12A1	COLGALT2	ICAM1	LAMB2	MMP9	TIMP2
ACTN1	COL13A1	COMP	ICAM2	LAMB3	MUSK	TLL1
ADAM10	COL14A1	CRTAP	ICAM3	LAMC1	NCAM1	TLL2
ADAM15	COL15A1	CTRB1	ICAM4	LAMC2	NCAN	TNC
ADAM17	COL16A1	CTRB2	ITGA1	LAMC3	NCSTN	TNN
ADAM8	COL17A1	CTSB	ITGA10	LOX	NID1	TNR
ADAM9	COL18A1	CTSD	ITGA11	LOXL1	NID2	TNXB
ADAMTS1	COL19A1	CTSG	ITGA2	LOXL2	NRXN1	TPSAB1
ADAMTS14	COL1A1	CTSK	ITGA2B	LOXL3	NTN4	TRAPPC4
ADAMTS16	COL1A2	CTSL	ITGA3	LOXL4	P3H1	TTR
ADAMTS18	COL20A1	CTSS	ITGA4	LRP4	P3H2	VCAM1
ADAMTS2	COL21A1	CTSV	ITGA5	LTBP1	P3H3	VCAN
ADAMTS3	COL22A1	DAG1	ITGA6	LTBP2	P4HB	VTN
ADAMTS4	COL23A1	DCN	ITGA7	LTBP3	PCOLCE	
ADAMTS5	COL24A1	DDR1	ITGA8	LTBP4	PCOLCE2	
ADAMTS8	COL25A1	DDR2	ITGA9	LUM	PDGFA	
ADAMTS9	COL26A1	DMD	ITGAD	MADCAM1	PDGFB	
AGRN	COL27A1	DMP1	ITGAE	MATN1	PECAM1	
ASPN	COL28A1	DSPP	ITGAL	MATN3	PLEC	
BCAN	COL2A1	DST	ITGAM	MATN4	PLG	
BGN	COL3A1	EFEMP1	ITGAV	MFAP1	PLOD1	
BMP1	COL4A1	EFEMP2	ITGAX	MFAP2	PLOD2	
BMP10	COL4A2	ELANE	ITGB1	MFAP3	PLOD3	
BMP2	COL4A3	ELN	ITGB2	MFAP4	PPIB	
BMP4	COL4A4	F11R	ITGB3	MFAP5	PRKCA	
BMP7	COL4A5	FBLN1	ITGB4	MMP1	PRSS1	
BSG	COL4A6	FBLN2	ITGB5	MMP10	PRSS2	
CAPN1	COL5A1	FBLN5	ITGB6	MMP11	PSEN1	
CAPNS1	COL5A2	FBN1	ITGB7	MMP12	PTPRS	
CASK	COL5A3	FBN2	ITGB8	MMP13	SDC1	
CASP3	COL6A1	FBN3	JAM2	MMP14	SDC2	
CD151	COL6A2	FGA	JAM3	MMP15	SDC3	
CD44	COL6A3	FGB	KDR	MMP16	SDC4	
CD47	COL6A5	FGF2	KLK2	MMP17	SERPINE1	
CDH1	COL6A6	FGG	KLK7	MMP19	SERPINH1	
CEACAM1	COL7A1	FMOD	KLKB1	MMP2	SPARC	
CEACAM6	COL8A1	FN1	LAMA1	MMP20	SPP1	
CEACAM8	COL8A2	FURIN	LAMA2	MMP24	TGFB1	
CMA1	COL9A1	GDF5	LAMA3	MMP25	TGFB2	
COL10A1	COL9A2	HAPLN1	LAMA4	MMP3	TGFB3	
COL11A1	COL9A3	HSPG2	LAMA5	MMP7	THBS1	

Supplemental Table S2. General characteristics of patients with SSc and healthy controls

Clinical variables	dc SSc<br>N = 26	HC<br>N = 16
Age, mean (SD), years	54.0 (13.5)	52.7 (17.6)
Male, n (%)	6 (23.1)	4 (25)
Race Black, n (%)	4 (15.4)	1 (6.25)
White, n (%)	22 (84.6)	15 (93.75)
Disease duration, mean (SD), years	2.7 (2.6)	
mRSS, mean (SD)	16.3 (10.6)	
ILD, n (%)	13 (50.0)	
Anti centromere positive, n (%)	1 (3.8)	
Anti Scl-70 positive, n (%)	5 (19.2)	
Anti RNA polymerase, n (%)	6 (23.1)	
Immunosuppressants, n (%)	25 (96.2)	

SSc, systemic sclerosis; dcSSc, diffuse cutaneous SSc; HC healthy controls; SD, standard deviation; mRSS, modified Rodnan skin score; ILD, interstitial lung disease.

**Supplementary Table S3: Statistics**		
Figure Number	Groups	P Value
Figure 1A	Normal vs. SSc	<0.0001
	Correlation SerpinE1 vs mRSS	0.0003
Figure 2A	Normal vs. dcSSc	0.0401
Figure 2B	SerpinE1	>0.9999
	ACTA2	0.0156
	COL1A1	0.0156
Figure 2D	Vehicle vs. MDI-2517 COL1A1	0.0064
	Vehicle vs. MDI-2517 SMA	0.0004
	Vehicle vs. MDI-2517 PAI-1	0.431
Figure 3B	Vehicle vs. MDI-2517	0.0147
Figure 3D	Vehicle vs. MDI-2517 Active PAI-1	0.0021
	Vehicle vs. MDI-2517 Total PAI-1	0.1229
Figure 4A	No treatment vs. Pirfenidone	0.4915
	No treatment vs. MMF	0.0573
	No treatment vs. MDI-2517	<0.0001
	Pirfenidone vs. MMF	0.5496
	Pirfenidone vs. MDI-2517	0.0015
	MMF vs. MDI-2517	0.0236
Figure 4B	Control Chow vs. Pirfenidone	0.9892
	Control Chow vs. MMF	<0.0001
	Control Chow vs. MDI-2517	<0.0001
	Pirfenidone vs. MMF	<0.0001
	Pirfenidone vs. MDI-2517	<0.0001
	MMF vs. MDI-2517	0.0583
Figure 4C	Vehicle vs. MDI-2517	0.0411
	Vehicle vs. Pirfenidone	0.4186
	Vehicle vs. MMF	0.042
	MDI-2517 vs. Pirfenidone	0.0074
	MDI-2517 vs. MMF	0.9907
	Pirfenidone vs. MMF	0.0076
Figure 4D	Vehicle vs. MDI-2517	0.0003
	Vehicle vs. Pirfenidone	0.4599
	Vehicle vs. MMF	0.0032
	MDI-2517 vs. Pirfenidone	0.0015
	MDI-2517 vs. MMF	0.2232
	Pirfenidone vs. MMF	0.0158

Figure 4E	Vehicle vs. MDI-2517	0.0056
	Vehicle vs. Pirfenidone	0.349
	Vehicle vs. MMF	0.3107
	MDI-2517 vs. Pirfenidone	0.0395
	MDI-2517 vs. MMF	0.0007
	Pirfenidone vs. MMF	0.0637
Figure 5A	Saline Vehicle vs. Saline MDI-2517	>0.9999
	Saline Vehicle vs. Bleo Vehicle	<0.0001
	Saline Vehicle vs. Bleo MDI-2517	0.0055
	Saline Vehicle vs. Bleo MMF	<0.0001
	Saline Vehicle vs. Bleo MMF+MDI-2517	0.0130
	Saline MDI-2517 vs. Bleo Vehicle	<0.0001
	Saline MDI-2517 vs. Bleo MDI-2517	0.0066
	Saline MDI-2517 vs. Bleo MMF	<0.0001
	Saline MDI-2517 vs. Bleo MMF+MDI-2517	0.0151
	Bleo Vehicle vs. Bleo MDI-2517	<0.0001
	Bleo Vehicle vs. Bleo MMF	0.4135
	Bleo Vehicle vs. Bleo MMF+MDI-2517	<0.0001
	Bleo MDI-2517 vs. Bleo MMF	0.0097
	Bleo MDI-2517 vs. Bleo MMF+MDI-2517	>0.9999
	Bleo MMF vs. Bleo MMF+MDI-2517	0.0275
Figure 5B	Control Chow vs. MDI-2517	0.0391
	Control Chow vs. MMF	0.9305
	Control Chow vs. MMF+MDI-2517	0.9868
	MDI-2517 vs. MMF	<0.0001
	MDI-2517 vs. MMF+MDI-2517	0.0033
	MMF vs. MMF+MDI-2517	0.4099
	Saline+Control Chow vs. Saline+MDI-2517	0.9928
Figure 6G	Vehicle vs. MDI-2517	0.0005
	Vehicle vs. MMF	0.9952
	Vehicle vs. MMF+MDI-2517	0.0017
	Vehicle vs. Vehicle	<0.0001
	Vehicle vs. MDI-2517	<0.0001
	MDI-2517 vs. MMF	0.0018
	MDI-2517 vs. MMF+MDI-2517	0.9952
	MDI-2517 vs. Vehicle	0.0097
	MDI-2517 vs. MDI-2517	0.0138
	MMF vs. MMF+MDI-2517	0.0038

	MMF vs. Vehicle	<0.0001
	MMF vs. MDI-2517	<0.0001
	MMF+MDI-2517 vs. Vehicle	0.0119
	MMF+MDI-2517 vs. MDI-2517	0.0151
	Vehicle vs. MDI-2517	0.9936
Figure 7G	Saline Vehicle vs. Saline MDI-2517	0.6761
	Saline Vehicle vs. Bleo Vehicle	<0.0001
	Saline Vehicle vs. Bleo MDI-2517	<0.0001
	Saline Vehicle vs. Bleo MMF	<0.0001
	Saline Vehicle vs. Bleo MMF+MDI-2517	<0.0001
	Saline MDI-2517 vs. Bleo Vehicle	<0.0001
	Saline MDI-2517 vs. Bleo MDI-2517	<0.0001
	Saline MDI-2517 vs. Bleo MMF	<0.0001
	Saline MDI-2517 vs. Bleo MMF+MDI-2517	<0.0001
	Bleo Vehicle vs. Bleo MDI-2517	<0.0001
	Bleo Vehicle vs. Bleo MMF	0.0131
	Bleo Vehicle vs. Bleo MMF+MDI-2517	<0.0001
	Bleo MDI-2517 vs. Bleo MMF	0.0259
	BleoMDI-2517 vs. Bleo MMF+MDI-2517	0.6106
	Bleo MMF vs. Bleo MMF+MDI-2517	0.0911
Figure 7H	Saline Vehicle vs. Saline MDI-2517	>0.9999
	Saline Vehicle vs. Bleo Vehicle	<0.0001
	Saline Vehicle vs. Bleo MDI-2517	0.2906
	Saline Vehicle vs. Bleo MMF	0.0224
	Saline Vehicle vs. Bleo MMF+MDI-2517	0.5965
	Saline MDI-2517 vs. Bleo Vehicle	<0.0001
	Saline MDI-2517 vs. Bleo MDI-2517	0.3008
	Saline MDI-2517 vs. Bleo MMF	0.0235
	Saline MDI-2517 vs. Bleo MMF+MDI-2517	0.6092
	Bleo Vehicle vs. Bleo MDI-2517	<0.0001
	Bleo Vehicle vs. Bleo MMF	0.0235
	Bleo Vehicle vs. Bleo MMF+MDI-2517	<0.0001
	Bleo MDI-2517 vs. Bleo MMF	0.6076
	Bleo MDI-2517 vs. Bleo MMF+MDI-2517	0.9972
	Bleo MMF vs. Bleo MMF+MDI-2517	0.4149
Figure 8A	Vehicle vs. MDI-2517	<0.0001
	Vehicle vs. Tiplaxtinin High	0.0995
	Vehicle vs. Tiplaxtinin Low	0.9308

	MDI-2517 vs. Tiplaxtinin High	<0.0001
	MDI-2517 vs. Tiplaxtinin Low	<0.0001
	Tiplaxtinin High vs. Tiplaxtinin Low	0.3008
Figure 8B	Vehicle vs. MDI-2517	<0.01
	Vehicle vs. Tiplaxtinin Low Dose	<0.01
	Vehicle vs. Tiplaxtinin High Dose	<0.01
	MDI-2517 vs. Tiplaxtinin Low Dose	<0.01
	MDI-2517 vs. Tiplaxtinin High Dose	<0.01
	Tiplaxtinin Low Dose vs. Tiplaxtinin High Dose	<0.01
Figure 9E	Vehicle vs. MDI-2517	0.001
	Vehicle vs. Tiplaxtinin High Dose	0.9427
	Vehicle vs. Tiplaxtinin Low Dose	0.9212
	MDI-2517 vs. Tiplaxtinin High Dose	0.0002
	MDI-2517 vs. Tiplaxtinin Low Dose	0.0002
	Tiplaxtinin High Dose vs. Tiplaxtinin Low Dose	0.9999
Figure 10E	Vehicle vs. MDI-2517	<0.0001
	Vehicle vs. Tiplaxtinin High Dose	0.0006
	Vehicle vs. Tiplaxtinin Low Dose	0.6916
	MDI-2517 vs. Tiplaxtinin High Dose	0.0001
	MDI-2517 vs. Tiplaxtinin Low Dose	<0.0001
	Tiplaxtinin High Dose vs. Tiplaxtinin Low Dose	0.0047
Figure 10F	Vehicle vs. MDI-2517	<0.0001
	Vehicle vs. Tiplaxtinin High Dose	0.1601
	Vehicle vs. Tiplaxtinin Low Dose	0.7807
	MDI-2517 vs. Tiplaxtinin High Dose	0.0001
	MDI-2517 vs. Tiplaxtinin Low Dose	<0.0001
	Tiplaxtinin High Dose vs. Tiplaxtinin Low Dose	0.7495
